# A Multiscale, Systems-Level, Neuropharmacological Model of Cortico-Basal Ganglia System for Arm Reaching Under Normal, Parkinsonian, and Levodopa Medication Conditions

**DOI:** 10.3389/fncom.2021.756881

**Published:** 2022-01-03

**Authors:** Sandeep Sathyanandan Nair, Vignayanandam Ravindernath Muddapu, V. Srinivasa Chakravarthy

**Affiliations:** ^1^Laboratory for Computational Neuroscience, Department of Biotechnology, Bhupat and Jyoti Mehta School of Biosciences, Indian Institute of Technology Madras, Chennai, India; ^2^Center for Complex Systems and Dynamics, Indian Institute of Technology Madras, Chennai, India

**Keywords:** levodopa, dopamine, basal ganglia, reinforcement learning, behavioural model, substantia nigra pars compacta, Parkinson's disease

## Abstract

In order to understand the link between substantia nigra pars compacta (SNc) cell loss and Parkinson's disease (PD) symptoms, we developed a multiscale computational model that can replicate the symptoms at the behavioural level by incorporating the key cellular and molecular mechanisms underlying PD pathology. There is a modelling tradition that links dopamine to reward and uses reinforcement learning (RL) concepts to model the basal ganglia. In our model, we replace the abstract representations of reward with the realistic variable of extracellular DA released by a network of SNc cells and incorporate it in the RL-based behavioural model, which simulates the arm reaching task. Our results successfully replicated the impact of SNc cell loss and levodopa (L-DOPA) medication on reaching performance. It also shows the side effects of medication, such as wearing off and peak dosage dyskinesias. The model demonstrates how differential dopaminergic axonal degeneration in basal ganglia results in various cardinal symptoms of PD. It was able to predict the optimum L-DOPA medication dosage for varying degrees of cell loss. The proposed model has a potential clinical application where drug dosage can be optimised as per patient characteristics.

## Introduction

Parkinson's disease is the second most prominent neurodegenerative disease after Alzheimer's (Gonzalez-Rodriguez et al., 2020; Marino et al., 2020; Muddapu and Chakravarthy, 2021). The onset of the disease is characterised by shaky movements, the rigidity of joints, unregulated movements, and even loss of smell (Morley and Duda, 2010; Fullard et al., 2017; Armstrong and Okun, 2020; Balestrino and Schapira, 2020; Goldman and Guerra, 2020; Marino et al., 2020). The major cause of Parkinson's disease (PD) is the death of dopaminergic neurons in substantia nigra pars compacta (SNc) (Michel et al., 2016; Surmeier, 2018; Muddapu et al., 2020a). Dopamine (DA) deficiency due to SNc cell loss manifest as the cardinal PD symptoms that include tremor, rigidity, bradykinesia, and postural imbalance (Bereczki, 2010; Poewe et al., 2017; Balestrino and Schapira, 2020). Epidemiological data from the United States alone indicates that there has been an exponential growth of people suffering from PD over the last few decades (Dorsey et al., 2018; Marras et al., 2018). However, the exact cause of this cell death is still not known. Various lines of investigation, experimental and computational, are in progress and hopefully, we will be able to narrow down the roots of this disease (Pissadaki and Bolam, 2013; Pacelli et al., 2015; Fu et al., 2018; Giguère et al., 2019; Muddapu et al., 2019, 2020a,b; Anilkumar et al., 2020; Gonzalez-Rodriguez et al., 2020; Muddapu and Chakravarthy, 2021). Understanding the cause and effect relationship between the underlying pathology and symptoms of any neurological disease has fundamental challenges since the roots of the disease are at the molecular and cellular level while the symptoms are seen at the behavioural level (Bakshi et al., 2019). Hence it is important to have a multi-scale model that spans molecular mechanisms to behavioural outputs. With this motivation in mind, we present a computational model that relates DA deficiency in PD to motor symptoms in ON and OFF conditions of medication. As an example of drug action, we simulate the effect of levodopa (L-DOPA) drug administration in our model.

The major ingredients of this computational modelling approach include a behavioural simulation of reaching task that replicates normal and parkinsonian movements, the basal ganglia cortico-motor circuitry that will drive the movements, a dopaminergic subsystem that modulates the control circuitry of the basal ganglia (BG), and pharmacological intervention of L-DOPA medication.

### Behavioural Simulation and the Cortico-Basal Ganglia Circuitry

Reaching movements is considered as one of the signatures of planned coordinated movement. Insights into the modelling approaches to these coordinated movements of the arm using the control feedback approach were inspired from previous modelling studies (Fitts, 1954; Morasso, 1981; Knill and Pouget, 2004; Körding and Wolpert, 2004; Todorov, 2004; Shadmehr and Krakauer, 2008). These studies didn't represent the neural correlates underlying the functionality. Later, neural correlates behind coordinated movements of arm (Doya, 1999; Nakahara et al., 2001; Hikosaka et al., 2002) and a reinforcement learning-based two-link arm model with kinetic parameters were developed (Izawa et al., 2004). In this study, we are trying the bridge the molecular level representation of dopamine to the behavioural level representation of motor movements.

### Basal Ganglia and Motor Learning

The interactions between the cortex and BG play a very important role in motor learning. It is through these interactions that the decision is made between two competing signals—one favouring the direction of movement and the other suppressing the movement. In order to facilitate this process, an action selection mechanism happens in BG subcortical structure, globus pallidus interna (GPi). The action selection mechanism of BG has been explored by various research groups (Gurney et al., 2001; Humphries et al., 2006; Bogacz and Gurney, 2007). Before the action selection takes place at GPi, the signal is forwarded to the GPi through distinct parallel projections from the striatum facilitated by DA receptor type 1 (D1) and DA receptor type 2 (D2) of medium spiny neurons (MSNs) that are modulated using the dopaminergic input from the SNc (Moustafa et al., 2016; Chakravarthy and Moustafa, 2018). In this study, we explored the role of DA in BG functioning and motor learning.

### Dopamine and BG Pathways

Dopaminergic input from the SNc neurons modulates the DA receptors present in the striatal neurons, the input nuclei of the BG differentially. The striatum consists of the D1 and D2 expressing MSNs that project via two different pathways. D1-MSN neurons project along the direct pathway, D2-MSNs project along the indirect pathway. The direct pathway projects directly to the output nuclei, GPi and substantia nigra pars reticulata (SNr), whereas the indirect pathway projects to the output nucleus, GPi, via globus pallidus externa (GPe), and subthalamic nucleus (STN). DA release from SNc neurons maintains the balance between activation of direct and indirect pathways. In order to understand the effect of DA deficiency as in PD conditions or the mechanism of DA replenishment by administration of L-DOPA, we need to understand DA synthesis, uptake, and release, which was explored in this study (Chakravarthy and Moustafa, 2018; Muddapu and Chakravarthy, 2021).

### Dopamine Deficiency and L-DOPA Medication

Dopamine (DA) deficiency due to SNc cell loss manifest as the cardinal PD symptoms that include tremor, rigidity, bradykinesia, and postural imbalance (Bereczki, 2010; Poewe et al., 2017; Balestrino and Schapira, 2020). L-DOPA is one of the first-line treatment methodologies for PD. The effect of L-DOPA medication on DA turnover processes in SNc terminal (Best et al., 2009; Reed et al., 2012) and its effects on neural systems of BG was modelled (Baston et al., 2016). However, the effect of L-DOPA medication at the behavioural level has not been explored. In this study, we explored the effect of DA deficiency and L-DOPA intervention on behavioural output.

In this paper, we present a multiscale model of the cortico-BG system to simulate arm reaching movements under normal, parkinsonian, and L-DOPA medication conditions. At the lowest level, the intracellular molecular pathways of SNc cells are modelled so as to capture dopamine synthesis, uptake, and release. At the next level, the BG circuitry is modelled using rate-coded neurons which are cast within the reinforcement learning framework with striatum acting as the neural correlate for critic and the direct and indirect pathways facilitating exploitation and exploration, respectively. At the highest level, arm reaching movements are modelled by a two-link arm model driven by a sensory-motor cortical loop.

This article is organised into multiple sections. Section Materials and methods describes the model architecture, equations, and methods. Here we discuss various functional loops that constitute the model and how they are interconnected. This section also covers the integration of pharmacological intervention. In section Results we showcase the results from the model starting with training the model, simulating the behaviour of a control subject, replicating the PD ON condition and some of the cardinal symptoms, assessing the performance in terms of reaching time, and verifying the effect of L-DOPA therapeutic intervention. The model results also gave an indicator of how to optimise the drug dosage. Section Discussion discusses the simulation results in detail and presents the potential future scope and based on that the conclusion derived is given in section Conclusions.

## Materials and Methods

**Figure 1:**
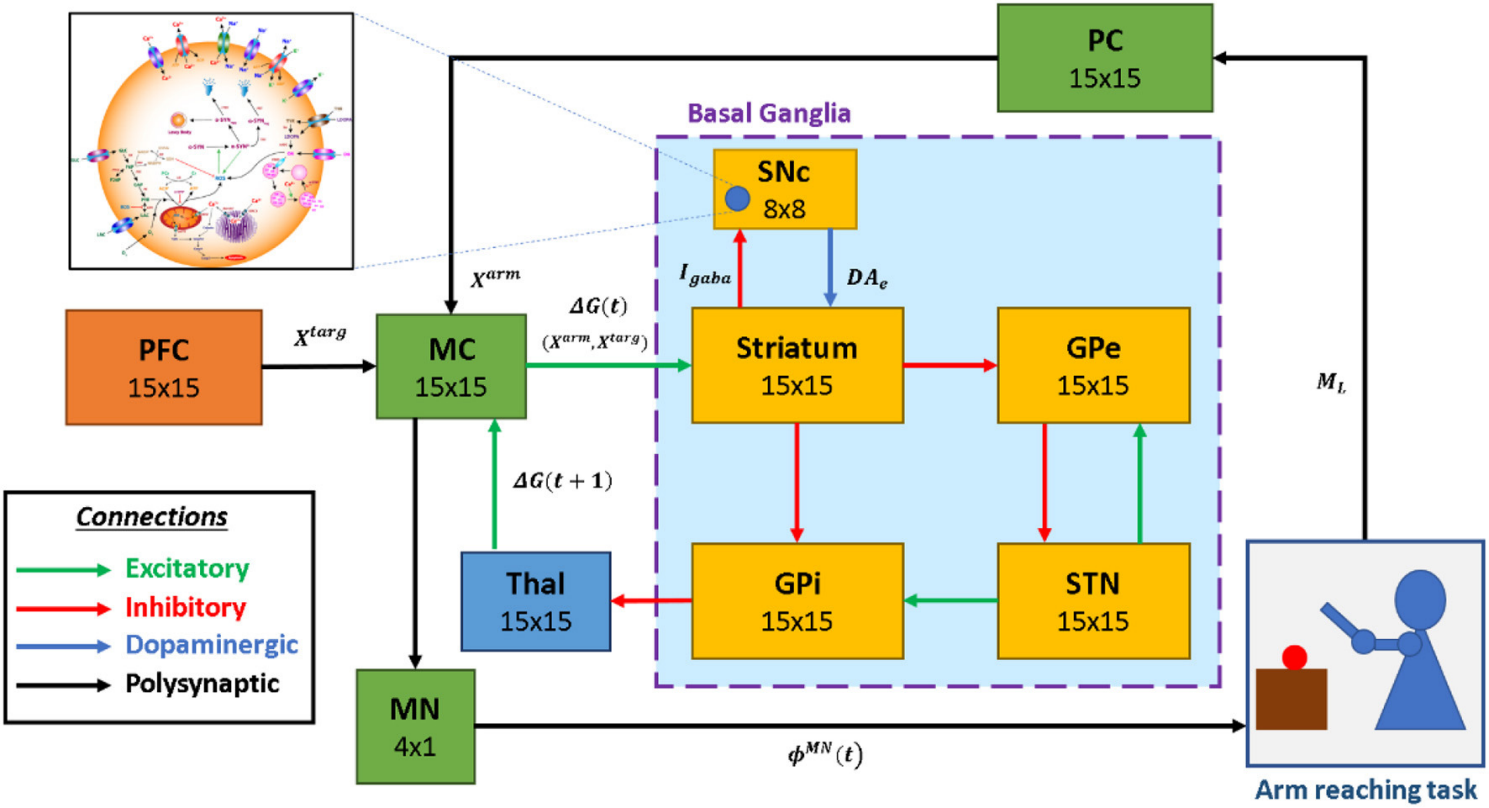
The model architecture of multiscale cortico-basal ganglia model for arm reaching. SNc, substantia nigra pars compacta; GPe, globus pallidus externa; GPi, globus pallidus interna; STN, subthalamic nucleus; Thal, thalamus; MC, motor cortex; MN, motor neuron; PC, proprioceptive cortex; PFC, the prefrontal cortex. *X*^*targ*^, the target position; *X*^*arm*^, the current arm position; ϕ^*MN*^, the motor neuron activations; *M*_*L*_, muscle lengths; *I*_*gaba*_, inhibitory GABAergic current; *DA*_*e*_, extracellular dopamine; *G*(*t*), the MC output; *G*(*t* + 1), the BG-derived activity of thalamus.

The proposed multiscale cortico-basal ganglia (MCBG) model was able to simulate the arm reaching in normal and Parkinsonian conditions which include some of the cardinal symptoms of PD (Figure 1). In addition, the effect of L-DOPA medication on arm reaching in PD condition was simulated (Supplementary Figure 1).

**Figure 2:**
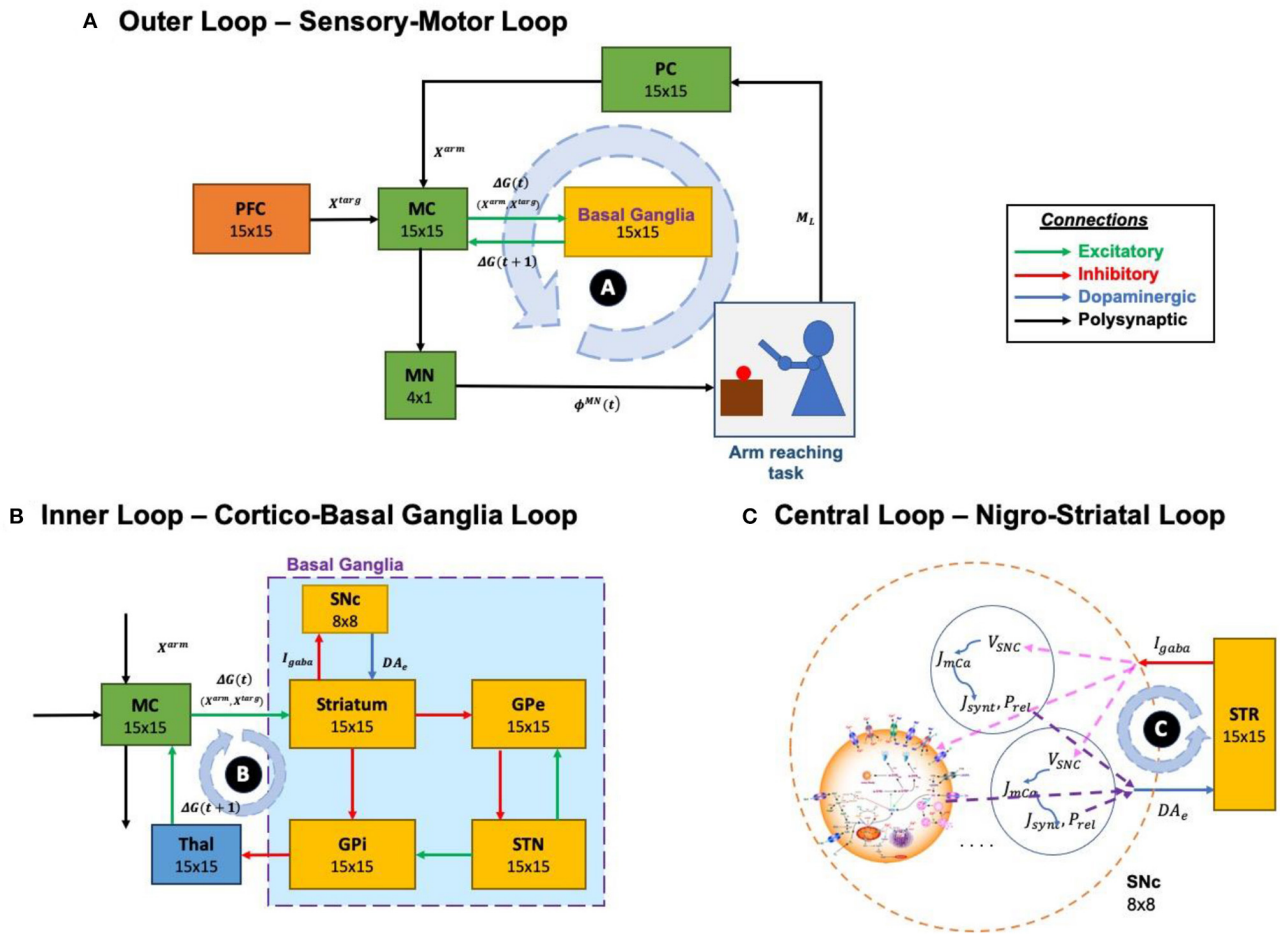
Different structural and functional loops of the proposed multiscale cortico-basal ganglia model. **(A)** Outer loop, sensory-motor loop; **(B)** Inner loop, cortico-basal ganglia loop; **(C)** Central loop, nigrostriatal loop. SNc, substantia nigra pars compacta; GPe, globus pallidus externa; GPi, globus pallidus interna; STN, subthalamic nucleus; STR, striatum; Thal, thalamus; MC, motor cortex; MN, motor neuron; PC, proprioceptive cortex; PFC, the prefrontal cortex. *X*^*targ*^, the target position; *X*^*arm*^, the current arm position; ϕ^*MN*^, the motor neuron activations; *M*_*L*_, muscle lengths; *I*_*gaba*_, inhibitory GABAergic current; *DA*_*e*_, extracellular dopamine; *G*(*t*), the MC output; *G*(*t* + 1), the BG-derived activity of thalamus; *V*_*SNc*_, the voltage membrane of SNc neuron; *J*_*m,Ca*_, the calcium flux of SNc neuron as a function of *V*_*SNc*_; *J*_*synt*_, the dopamine synthesis flux as a function of calcium; *P*_*rel*_, the probability release of dopamine extracellularly as a function of calcium.

The proposed model can be broadly described in three parts. (i) Outer loop—motor-sensory loop, (ii) Inner loop—cortico-basal ganglia loop, and (iii) Central loop—nigrostriatal loop (Figure 2). The outer loop consists of the motor cortex (MC), motor neurons (MNs), arm, proprioceptive cortex (PC), and prefrontal cortex (PFC). The inner loop consists of MC, thalamus, and BG nuclei comprised of the striatum, GPi, GPe, and STN. The central loop consists of striatum and SNc, which plays an important role in simulating PD conditions, where nigrostriatal and nigrosubthalamic pathways are affected by SNc cell loss. For L-DOPA medication, a pharmacokinetic module was formulated where input will be L-DOPA dosage given to the PD patient and output will be the amount of DA released in the striatum during the medication. The subsequent sections describe the dynamics involved in each of these three loops.

### Outer Loop—Sensory-Motor Loop

The functional pathway of the outer loop is shown in Figure 2A. The outer loop consists of a two-link arm model driven by MNs. MNs receive motor commands from MC. The end effector position of the arm is sensed by PC and it forwards the signal to MC, which receives signals from PFC and BG. MC issues the motor commands based on the integration of incoming signals.

#### Arm Model

The kinetic model of the two-joint arm simulates the movement of the arm in two-dimensional space (Izawa et al., 2004; Zadravec and Matjačić, 2013; Supplementary Figure 2). Each joint (shoulder and elbow) is controlled by an agonist (*Ag*) and antagonist (*An*) muscle pair where the shoulder joint is controlled by anterior deltoid (shoulder flexor, *M*_1_) and posterior deltoid (shoulder extensor, *M*_2_) and elbow joint is controlled by brachialis (elbow flexor, *M*_3_) and triceps brachii (elbow extensor, *M*_4_) (Jagodnik and van den Bogert, 2010). The activations to these muscle groups (ϕ^*MN*^) are transformed into joint angles for both shoulder and the elbow as follows,


(1) $$\begin{array}{l} \theta_{S}^{JA}\left(t\right)=\left(\phi_{S_{Ag}}^{MN}\left(t\right)-\phi_{S_{An}}^{MN}\left(t\right)\right)\frac{\pi }{2}+\frac{\pi }{2} \end{array}$$

(2) $$\begin{array}{l} \theta_{E}^{JA}\left(t\right)=\left(\phi_{E_{Ag}}^{MN}\left(t\right)-\phi_{E_{An}}^{MN}\left(t\right)\right)\frac{\pi }{2}+\frac{\pi }{2} \end{array}$$

where, $\theta_{S}^{JA}$ and $\theta_{E}^{JA}$ are the joint angles of shoulder and elbow with respect to the x-axis (Supplementary Figure 1) and shoulder-length (*l*_*S*_), respectively, in two-dimensional space, $\phi_{S_{Ag}}^{MN}$ is the muscle activation of shoulder agonist muscle, $\phi_{S_{An}}^{MN}$ is the muscle activation of shoulder antagonist muscle, $\phi_{E_{Ag}}^{MN}$ is the muscle activation of elbow agonist muscle, and $\phi_{E_{An}}^{MN}$ is the muscle activation of the elbow antagonist muscle.

The coverage of the arm in two-dimensional space is controlled by these joint angles. The joint angles are used to calculate the muscle lengths for both shoulder and elbow as given below.


(3) $$\begin{array}{l} \mu_{Ag}^{S}\left(t\right)=\sqrt{a_{S}^{2}+b_{S}^{2}+2a_{S}b_{S}\cos\left(\theta_{S}^{JA}\right)} \end{array}$$

(4) $$\begin{array}{l} \mu_{An}^{S}\left(t\right)=\sqrt{a_{S}^{2}+b_{S}^{2}-2a_{S}b_{S}\cos\left(\theta_{S}^{JA}\right)} \end{array}$$

(5) $$\begin{array}{l} \mu_{Ag}^{E}\left(t\right)=\sqrt{a_{E}^{2}+b_{E}^{2}+2a_{E}b_{E}\cos\left(\theta_{E}^{JA}\right)\;} \end{array}$$

(6) $$\begin{array}{l} \mu_{AN}^{E}\left(t\right)=\sqrt{a_{E}^{2}+b_{E}^{2}-2a_{E}b_{E}\cos\left(\theta_{E}^{JA}\right)} \end{array}$$

where, $\mu_{Ag}^{S}$, $\mu_{An}^{S}$, $\mu_{Ag}^{E}$, and $\mu_{An}^{E}$ are the agonist and antagonist muscle lengths of shoulder and elbow, respectively, *a*_*S*_ is the distance between the shoulder join centre and *M*_1_ or *M*_2_ moment lever, *b*_*S*_ is the distance between the shoulder joint centre and *M*_1_ or *M*_2_ moment lever, *a*_*E*_ is the distance between elbow joint centre and *M*_3_ or *M*_4_ moment lever, and *b*_*E*_ is the distance between elbow joint centre and *M*_3_ or *M*_4_ moment lever.

Using these muscle lengths in the form of a four-dimensional vector $\left(M_{L}=\left[\mu_{Ag\;}^{S}\mu_{An\;}^{S}\mu_{Ag}^{E}{\;\mu }_{An}^{E}\right]\right)$, a sensory (proprioceptive) map of the arm was generated. The end effector position of the arm $\left(X^{arm}=\left[x_{1}^{arm}\;x_{2}^{arm}\right]\right)$ in the two-dimensional space is calculated as,


(7) $$\begin{array}{l} x_{1}^{arm}=\left(l_{S}-a_{S}\right)\cos\left(\theta_{S}^{JA}\right)+l_{E}\cos\left(\theta_{S}^{JA}+\theta_{E}^{JA}\right) \end{array}$$

(8) $$\begin{array}{l} x_{2}^{arm}=\left(l_{S}-a_{S}\right)\sin\left(\theta_{S}^{JA}\right)+l_{E}\sin\left(\theta_{S}^{JA}+\theta_{E}^{JA}\right) \end{array}$$

(9) $$\begin{array}{l} M_{L}=\left[\mu_{Ag}^{S}\;\mu_{An}^{S}\;\mu_{Ag}^{E}\;\mu_{An}^{E}\right] \end{array}$$

where, $\theta_{S}^{JA}$ and $\theta_{E}^{JA}$ are the joint angles of shoulder and elbow with respect to the x-axis (Supplementary Figure 1) and shoulder-length (*l*_*S*_), respectively, in two-dimensional space, *l*_*S*_ is the distance between the shoulder joint centre (*S*) and elbow joint centre (*E*), *l*_*E*_ is the distance between the elbow joint centre (*E*) and end effector (*H*), *a*_*S*_ is the distance between the shoulder joint centre and *M*_1_ or *M*_2_ moment lever, $\mu_{Ag}^{S}$, $\mu_{An}^{S}$, $\mu_{Ag}^{E}$, and $\mu_{An}^{E}$ are the agonist (*M*_1_ or *M*_3_) and antagonist (*M*_2_ or *M*_4_) muscle lengths of shoulder and elbow, respectively.

#### Proprioceptive Cortex

The proprioceptive cortex (PC) is modelled as a self-organising map (SOM) (Kohonen, 2001) of size *N*_*PC*_ *x N*_*PC*_ where the sensory map of the arm was generated. Using muscle length vector (*M*_*L*_(*t*)) from the arm model (Equation 9) as a feature vector, PC SOM was trained. The activation of a single node (*i, j*) in the PC SOM is given as,


(10) $$\begin{array}{l} U_{ij}^{PC}\left(t\right)=\exp\left(\frac{-{\left\|M_{L}\left(t\right)-W_{PC,ij}\right\|}^{2}}{\sigma_{PC}^{2}}\right) \end{array}$$

where, *W*_*PC,i*_ is the weight of the connection between the muscle length vector and *i*^*th*^ the neuron of the two-dimensional PC network, *M*_*L*_ is the muscle length vector and σ_*PC*_ is the width of the Gaussian response of PC SOM.

#### The Prefrontal Cortex

The prefrontal cortex (PFC) encodes the goal position where, in real-time, the goal information is formed using the visual sensory feedback, which is passed on to the frontal areas. In our current model, we fix the goal or target position and denote it by *X*^*targ*^. The motor command initially is driven by the PFC as the PFC specifies the goal to be reached. Similar to the PC, the PFC SOM is trained using the target position vector as a feature vector. The input features of the PFC are the spatial locations where the arm can possibly reach in the two-dimensional space. The target locations that produce the activation in the PFC network is given as.


(11) $$\begin{array}{l} U_{ij}^{PFC}\left(t\right)=\exp\left(\frac{-{\left\|X^{targ}\left(t\right)-W_{PFC,ij}\right\|}^{2}}{{\sigma_{PFC}}^{2}}\right) \end{array}$$

where, *W*_*PFC,ij*_ are the weight of the connection between the target position vector and (*i, j*)^*th*^ the neuron of the two-dimensional PFC network, *X*^*targ*^ is the target position and σ_*PFC*_ is the width of the Gaussian response of PFC SOM.

#### Motor Cortex

Motor cortex (MC) is modelled as a combination of SOM and continuous attractor neural network (CANN) (Trappenberg, 2005) of size *N*_*MC*_ *x N*_*MC*_. This type of architecture of MC is used to account for two distinct characteristics of cortical areas *viz*., low dimensional representation of input space and dynamics based on the connectivity in these cortical regions. A dynamic model like CANN is employed to facilitate the integration of multiple afferent inputs received from the PC, the BG, and PFC. The output activity of the MC CANN (*G*_*MC*_) is defined by,


(12) $$\begin{array}{l} G_{MC}\left(t\right)=\frac{g_{MC}^{2}}{1+\left(\frac{2\pi }{N_{MC}^{2}}\right)b_{MC}\sum g_{MC}^{2}} \end{array}$$

where, *g*_*MC*_ is the internal state of MC CANN, *N*_*MC*_ is the size of MC network, *b*_*MC*_ is the constant term.

The internal state of the MC CANN (*g*_*MC*_) is given by,


(13) $$\begin{array}{l} \tau_{MC}\frac{dg_{MC}}{dt}=-g_{MC}+W_{MC}^{C}\;⊗G_{MC}+I_{MC} \end{array}$$

where, ${\;}_{MC}^{C}$ is the weight kernel representing lateral connectivity in MC CANN, which determines the local excitation/global inhibition dynamics, *G*_*MC*_ is the output activity of MC CANN, *I*_*MC*_ is the total input coming into MC CANN from PC, PFC, and BG and ⊗ represents the convolutional operation.

##### Lateral Connections in MC

The lateral connectivity in the MC CANN is characterised by short-range (local) excitation and long-range (global) inhibition whose dynamics are defined by the weight kernel $\left(W_{MC}^{C}\right)$ is given by,


(14) $$\begin{array}{l} W_{MC,i,j}^{C}=A_{lat}^{C}\exp\left(\frac{-{\left\|\left(i_{MC}-i_{h}\right)+\left(j_{MC}-j_{h}\right)\right\|}^{2}}{2{\left(\sigma_{lat}^{C}\right)}^{2}}\right)-K^{C} \end{array}$$

where, [*i*_*MC*_, *j*_*MC*_] are the location of the nodes in MC, [*i*_*h*_, *j*_*h*_] corresponds to the central node, $A_{lat}^{C}$ is the strength of the excitatory connections, *K*^*C*^ is the global inhibition constant and $\sigma_{lat}^{C}$ is the radius of the excitatory connections.

##### Total Inputs Into MC

The total input (*I*_*MC*_) coming into MC CANN from PC (information about the current position of the arm), PFC (information about target position), and BG (error feedback signal) is given by,


(15) $$\begin{array}{l} I_{MC}\left(t\right)=A_{PC}G_{PC}\left(t\right)+A_{PFC}G_{PFC}\left(t\right)+A_{BG}G_{BG}\left(t\right) \end{array}$$

where, *A*_*PC*_, *A*_*PFC*_, *A*_*BG*_ are the respective gains of PC, PFC, and BG, *G*_*PC*_, *G*_*PFC*_, *G*_*BG*_ are the output activities of PC-derived SOM part of MC, PFC-derived activation part of MC, and BG-derived network activity of thalamus.

PC activity is used to generate low-level feature maps in MC using the SOM algorithm. The activation of the (*i, j*)^*th*^node in the SOM part of the MC (*G*_*PC,ij*_) is given as,


(16) $$\begin{array}{l} G_{PC,ij}\left(t\right)=\exp\left(\frac{-{\left\|U^{PC}\left(t\right)-W_{MC,ij}\right\|}^{2}}{\sigma_{MC}^{2}}\right) \end{array}$$

where, *U*^*C*^ is the output activity of PC SOM network, *W*_*MC,i*_ is the weight of the connection between the PC SOM network and *i*^*th*^ neuron of the two-dimensional MC SOM network, and σ_*MC*_ is the width of the Gaussian response of MC SOM.

The input from PFC to MC (*G*_*PFC*_) is the product of weight matrix (*W*_*PFC*→*MC*_) and the output activity of PFC SOM is given by,


(17) $$\begin{array}{l} G_{PFC}\left(t\right)=W_{PFC\to MC}*U^{PFC}\left(t\right) \end{array}$$

where, *U*^*PFC*^ is the output activity of the PFC SOM network, *W*_*PFC*→*MC*_ is the weight matrix between PFC and MC.

In an earlier line of modelling studies, we have shown that the classical Go-NoGo interpretation of the functional anatomy of the BG must be expanded to Go-Explore-NoGo, in view of the putative role of the Indirect Pathway in exploration (Sridharan et al., 2006; Chakravarthy and Balasubramani, 2014). This series of models had resulted in the so-called Go-Explore-NoGo policy, which refers to a stochastic hill-climbing performed on the value function computed inside the BG (Magdoom et al., 2011). When the arm reaches the target (ϵ < 0.1), the connections between the PFC and MC are trained by,


(18) $$\begin{array}{l} W_{PFC\to MC}=\eta_{PFC\to MC}\left(G_{targ}^{MC}\left(t\right)-G_{PFC}^{MC}\left(t\right)\right)U^{PFC}\left(t\right) \end{array}$$

where, η_*PFC*→*MC*_ is the learning rate between PFC and MC, $G_{targ}^{MC}$ is the MC activation required for the arm to reach the target, and $G_{PFC}^{MC}\left(G_{PFC}\right)$ is the MC activation due to PFC.

#### Motor Neurons

The output activity of MC CANN projects to the MN layer which consists of four MNs that drives four muscles of the arm whose activation is given by,


(19) $$\begin{array}{l} \phi^{MN}=A_{MN}W_{MC\to MN}G_{MC}\left(t\right) \end{array}$$

where, *A*_*MN*_ is the gain of MN, *W*_*MC*→*MN*_ is the weight matrix between MC CANN and MN layer, and *G*_*MC*_ is the output activity of MC CANN.

To close the sensory-motor loop, we perform a comparison with the initial activation to the MN layer that was used to produce desired activation $\phi_{D}^{MN}\left(t\right)$. The weights between the MN and MC are trained in a supervised manner by comparing the network-derived MN activation ϕ^*MN*^(*t*) to the desired activation $\phi_{D}^{MN}\left(t\right)$. This gives a loop that is consistent in mapping the external arm space to the neuronal space and vice. The connection between MC and MN is trained by,


(20) $$\begin{array}{l} \Delta W_{MC\to MN}=\eta_{MC\to MN}\left(\phi_{D}^{MN}\left(t\right)-\phi^{MN}\left(t\right)\right)G_{MC}\left(t\right) \end{array}$$

where, η_*MC*→*MN*_ is the learning rate between MC and MN, $\phi_{D}^{MN}$ is the desired MN activation required for the arm to reach the target and ϕ^*MN*^ is the network-derived MN activation due to MC, and *G*_*MC*_ is the output activity of MC CANN. The training schema for the outer loop (sensory-motor loop) is described in Supplementary Information.

### Inner Loop—Cortico-Basal Ganglia Loop

The functional pathway of the inner loop is shown in Figure 2B. The inner loop consists of MC, BG, and thalamus. MC receives information from BG via the thalamus. MC sends information to BG based on the integration of incoming signals received from PFC (target goal position, *X*^*targ*^), PC (current end-effector position of the arm, *X*^*arm*^) and BG (via thalamus, error feedback signal, *G*_*BG*_).

#### Basal Ganglia

Basal ganglia (BG) consists of the striatum, GPe, GPi, STN, and SNc. The output signal from BG provides the necessary control for the arm to reach the target by modulating the MC activity. The output of the MC is as given in Equation 12.

##### Value Computation and Stochastic Hill Climbing

The signal from the PC contains information about the current end-effector position of the arm (*X*^*arm*^) whereas the signal from PFC contains the target goal position (*X*^*targ*^). These two signals are combined in the BG to form a value function, *V*^*arm*^(*t*), that represents the error between the desired and the actual positions of the hand as,


(21) $$\begin{array}{l} V^{arm}\left(t\right)=\exp\left(\frac{-{\left\|X^{targ}-X^{arm}\right\|}^{2}}{\sigma_{V}^{2}}\right) \end{array}$$

where, *X*^*targ*^ is the target goal position, *X*^*arm*^ is the current end-effector position of the arm, σ_*V*_ is the spatial range over which the value function is sensitive for that particular target.

The output of the BG performs a stochastic hill-climbing over the value function (Chakravarthy and Moustafa, 2018; Narayanamurthy et al., 2019) and drives the MC to facilitate the arm in reaching the target. The value difference (δ_*V*_) which is computed by comparing the current and previous values is given as,


(22) $$\begin{array}{l} \delta_{V}=V^{arm}\left(t\right)-V^{arm}\left(t-1\right) \end{array}$$

where, *V*^*arm*^(*t*) is the current value and *V*^*arm*^(*t* − 1) is the previous value.

Based on this value difference signal (δ_*V*_), the striatum will send the inhibitory GABAergic current (*I*_*gaba*_) to the SNc neurons while the SNc neurons will in turn release dopamine into the extracellular space (*DA*_*e*_), which is absorbed by the striatum. *DA*_*e*_ is transformed into $\delta_{V}^{SNc}$. $\delta_{V}^{SNc}$ modulates the selection of direct and indirect pathways in the BG. The dynamics between the striatum and the SNc are described in greater detail in the subsequent section, “The Central Loop.”

##### Action Selection

*Striatum*: The resultant $\delta_{V}^{SNc}$ acts as a modulatory signal on D1R-MSNs and D2R-MSNs of the striatum, which processes the input signal, Δ*G*_*MC*_(*t*), and send outputs *y*_*D*1_ & *y*_*D*2_ via direct and indirect pathways, respectively.


(23) $$\begin{array}{l} y_{D1}=\lambda_{D1}W_{CTX\to D1}\Delta G_{MC} \end{array}$$

(24) $$\begin{array}{l} y_{D2}=\lambda_{D2}W_{CTX\to D2}\Delta G_{MC} \end{array}$$

(25) $$\begin{array}{l} \lambda_{D1}=\frac{1}{1+\exp\left(-a_{D1}\left(\delta_{V}^{SNc}-\theta_{D1}\right)\right)} \end{array}$$

(26) $$\begin{array}{l} \lambda_{D2}=\frac{1}{1+\exp\left(-a_{D2}\left(\delta_{V}^{SNc}-\theta_{D2}\right)\right)} \end{array}$$

where, λ_*D*1_ and λ_*D*2_ represent the effect of dopamine (value difference) on the D1 and D2 MSNs, respectively, *W*_*CTX*→*D*1_ and *W*_*CTX*→*D*2_ represent connections between cortex and D1 MSNs and cortex and D2 MSNs, respectively, Δ*G*_*MC*_ is the output activity of MC, $\delta_{V}^{SNc}$ is the SNc-derived value difference, θ_*D*1_ and θ_*D*2_ are the thresholds of the D1 and D2 MSNs, respectively, *a*_*D*1_ and *a*_*D*2_ are the sigmoidal gains of the D1 and D2 MSNs, respectively. Since *a*_*D*1_ = −*a*_*D*2_, the activation of direct and indirect pathways depends on the $\delta_{V}^{SNc}$ such that when $\delta_{V}^{SNc}$ is positive (negative) the direct (indirect) pathway is selected. Note that the λ_*D*1_ and λ_*D*2_ parameters in Equations (25–26) are dependent only on $\delta_{V}^{SNc}$ (“tonic dopamine”) and not on its temporal derivative (“phasic dopamine”).

*STN-GPe subsystem*: In the indirect pathway, D2 MSNs of the striatum project to the GPe, where *y*_*D*2_ influences GPe neural dynamics, which in turn influences STN neural dynamics. STN-GPe forms a loop with inhibitory projections from GPe to STN and excitatory projections from STN to GPe. Such excitatory-inhibitory pairs of neuronal pools have been shown to exhibit limit cycle oscillations (Gillies et al., 2002) which was modelled as coupled Van der Pol oscillator (Kawahara, 1980). The dynamics of the STN-GPe system is defined as,


(27) $$\begin{array}{l} \tau_{GPe}\frac{dx_{GPe}}{dt}=-x_{GPe}\\ \;+\varepsilon_{g}\sum \sum W_{glat}x_{GPe}\\ \;+w_{sg}y_{STN}+y_{D2} \end{array}$$

(28) $$\begin{array}{l} \tau_{STN}\frac{dx_{STN}}{dt}=-x_{STN}\\ \;+\varepsilon_{s}\sum \sum W_{slat}y_{STN}-w_{gs}x_{GPe} \end{array}$$

(29) $$\begin{array}{l} y_{STN}=\tanh\left(\lambda_{STN}x_{STN}\right) \end{array}$$

where, *x*_*GPe*_ and *x*_*TN*_ are the internal states of GPe and STN neurons, respectively, *y*_*STN*_ is the output of STN neuron, ε_*g*_ and ε_*s*_ are the strengths of lateral connections in GPe and STN networks, respectively, *W*_*glat*_ and *W*_*slat*_ are weight kernels representing lateral connectivity in GPe and STN networks, respectively, *y*_*D*2_ is the output of D2 MSN, τ_*GPe*_ and τ_*STN*_ are the time constants of GPe and STN, respectively, *w*_*sg*_ is the connection strength from STN to GPe, *w*_*gs*_ is the connection strength from GPe to STN, and λ_*STN*_ is the parameter which controls the slope of the sigmoid in STN.

*Lateral Connections in STN-GPe:* The lateral connectivity in STN or GPe network is modelled as Gaussian neighbourhood (Muddapu et al., 2019) which is defined by the weight kernel (*W*_*glat*/*slat*_) as,


(30) $$\begin{array}{l} W_{i,j,k,l}^{glat/slat}=\exp\left(-\frac{d_{i,j,k,l}^{2}}{{\left(\sigma_{lat}^{g/s}\right)}^{2}}\right) \end{array}$$

(31) $$\begin{array}{l} d_{i,j,k,l}^{2}={\left(i_{g/s}-k_{g/s}\right)}^{2}+{\left(j_{g/s}-l_{g/s}\right)}^{2} \end{array}$$

where, $d_{i,j,k,l}^{2}$ is the distance of neuron (*i, j*) from a centre neuron (*k, l*), $\sigma_{lat}^{g/s}$ is the spread of the lateral connections for GPe or STN network. The detailed analysis of the STN-GPe subsystem is described in section STN-GPe Dynamics of the Supplementary Material.

*GPi:* The signals arriving from D1 MSN (*y*_*D*1_) and STN (*y*_*STN*_) via direct and indirect pathways, respectively, combines in GPi which is defined as,


(32) $$\begin{array}{l} y_{GPi}=A_{D1}y_{D1}-A_{D2}y_{STN} \end{array}$$

where, *y*_*D*1_ is the output of D1 MSN via direct pathway, *y*_*STN*_ is the output of STN via indirect pathway, *A*_*D*1_ and *A*_*D*2_ are the gains of direct and indirect pathways, respectively.

#### Thalamus

The combined inputs (*y*_*GPi*_) at GPi from direct (*y*_*D*1_) and indirect (*y*_*STN*_) pathways are then passed on to the thalamus. To integrate and philtre the information from the GPi output, the thalamus was modelled as a CANN which is defined as,


(33) $$\begin{array}{l} G_{thal}\left(t\right)=\frac{g_{thal}^{2}}{1+\left(\frac{2\pi }{N_{thal}^{2}}\right)b_{thal}\sum g_{thal}^{2}} \end{array}$$

where, *g*_*thal*_ is the internal state of thalamus CANN, *N*_*thal*_ is the size of thalamus network, *b*_*thal*_ is the constant term.

The internal state of the thalamus CANN (*g*_*thal*_) is given by,


(34) $$\begin{array}{l} \tau_{thal}\frac{dg_{thal}}{dt}=-g_{thal}+W_{thal}^{C}\;⊗G_{thal}+I_{BG} \end{array}$$

(35) $$\begin{array}{l} I_{BG}=y_{GPi} \end{array}$$

(36) $$\begin{array}{l} G_{BG}=G_{thal} \end{array}$$

where, $W_{thal}^{C}$ is the weight kernel representing lateral connectivity in thalamus CANN, which determines the local excitation/global inhibition dynamics, *G*_*thal*_ is the output activity of thalamus CANN, *I*_*BG*_ is the total input coming into thalamus CANN from BG, *y*_*GPi*_ is the output of GPi, *G*_*BG*_ is the BG-derived network activity of the thalamus, and ⊗ represents the convolution operation.

### Central Loop—Nigro-Striatal Loop

The functional pathway of the central loop is as represented in Figure 2C. The central loop consists of the striatum (the input nucleus of BG) and SNc. SNc projects to the striatum via dopaminergic axons (*DA*_*e*_) and striatum in turn projects to SNc via inhibitory GABAergic axons (*I*_*gaba*_). Based on the sensory feedback signal received from the PC (*X*^*arm*^) and the target information from the PFC (*X*^*targ*^), the striatum performs value computation (*V*^*arm*^). Based on the values from current (*V*^*arm*^(*t*)) and previous (*V*^*arm*^(*t* − 1)) instants, the value difference (error, δ_*V*_) is computed. Based on the value difference (δ_*V*_), appropriate amount of GABAergic current (*I*_*gaba*_) is sent to SNc, which in turn releases dopamine into the striatum (*DA*_*e*_) accordingly.

#### SNc

##### SNc Neuron (Soma)

The detailed single-compartmental biophysical model of the SNc neuron is adopted from Muddapu and Chakravarthy (2021). The model incorporates all the essential molecular level mechanisms such as ion channels, active pumps, ion exchangers, dopamine turnover processes, etc.

Based on the value difference signal (δ_*V*_), the inhibitory GABAergic current (*I*_*gaba*_), flows from the striatum to SNc. *I*_*gaba*_ along with excitatory glutamatergic current (*I*_*nmda*/*ampa*_) contributes to the overall synaptic input current flux (*J*_*syn*_) to the SNc neurons. *J*_*syn*_ regulates the membrane voltage of the SNc along with the sodium, calcium, and potassium fluxes as given by,


(37) $$\begin{array}{l} \frac{d\left(V_{SNc}\right)}{dt}=\frac{F*vol_{cyt}}{C_{snc}*AR_{pmu}}*\left[\;J_{m,Na}+2*J_{m,Ca}+J_{m,K}+J_{syn}\right] \end{array}$$

(38) $$\begin{array}{l} J_{syn}=-\frac{1}{F*vol_{cyt}}*\left(I_{gaba}+\;I_{nmda/ampa}\right) \end{array}$$

(39) $$\begin{array}{l} I_{gaba}=F\left(\delta_{V}\right) \end{array}$$

where, *F* is the Faraday's constant, *C*_*snc*_ is the SNc membrane capacitance, *vol*_*cyt*_ is the cytosolic volume, *AR*_*pmu*_ is the cytosolic area, *J*_*m,Na*_ is the sodium membrane ion flux, *J*_*m,Ca*_ is the calcium membrane ion flux, *J*_*m,K*_ is the potassium membrane ion flux, *J*_*syn*_ is the overall input current flux, δ_*V*_ is the value difference, *I*_*gaba*_ is the inhibitory GABAergic current flux, and *I*_*nmda*/*ampa*_ is the excitatory glutamatergic (NMDA/AMPA) current flux.

The membrane voltage of SNc (*V*_*SNc*_) regulates the calcium membrane ionic flux which results in calcium oscillations inside SNc neuron. The calcium membrane ionic flux (*J*_*m,Ca*_) is given by,


(40) $$\begin{array}{l} J_{m,Ca}=-\frac{1}{z_{Ca}*F*vol_{cyt}}*\left(I_{CaL}+\;{2*I}_{pmca}-{2*I}_{NaCaX}\right) \end{array}$$

where, *F* is the Faraday's constant, *z*_*Ca*_ is the valence of calcium ion, *vol*_*cyt*_ is the cytosolic volume, *I*_*CaL*_ is the L-type calcium channel current, *I*_*pmca*_ is the ATP-dependent calcium pump current, and *I*_*NaCaX*_ is the sodium-potassium exchanger current.

The intracellular calcium oscillation or dynamics ([*Ca*_*i*_]) is defined as,


(41) $$\begin{array}{l} \frac{d\left[Ca_{i}\right]}{dt}=J_{m,Ca}-J_{calb}-4*J_{cam} \end{array}$$

where, *J*_*m,Ca*_ is the flux of calcium ion channels, *J*_*calb*_ is the calcium buffering flux by calbindin, and *J*_*cam*_ is the calcium buffering flux by calmodulin. A detailed description of the SNc neuron is provided in section Biophysical Model of SNc of the Supplementary Material.

##### SNc Terminal

The three-compartmental biochemical model of the SNc terminal is adopted from Muddapu and Chakravarthy (2021). The SNc terminal model incorporates all the necessary molecular-level mechanisms of the dopamine turnover process such as synthesis, packing, release, and reuptake.

*Calcium-Dependent Dopamine Release:* Dopamine (DA) synthesis and release by SNc terminal depend on calcium oscillations. The flux of dopamine release (*J*_*rel*_) from the SNc terminal is given by,


(42) $$\begin{array}{l} J_{rel}=\psi *n_{RRP}*P_{rel}\left(\left[Ca_{i}\right]\right) \end{array}$$

where, [*Ca*_*i*_] is the intracellular calcium concentration in the SNc terminal, *P*_*rel*_ is the release probability as a function of intracellular calcium concentration, *n*_*RRP*_ is the average number of readily releasable vesicles, and ψ is the average release flux per vesicle within a single synapse.

*Calcium-Dependent Dopamine Synthesis:* The flux of calcium-dependent dopamine synthesis is defined as,


(43) $$\begin{array}{l} V_{synt}\left(Ca_{i}\right)={\bar{V}}_{synt}*\frac{{\left[Ca_{i}\right]}^{4}}{K_{synt}^{4}+{\left[Ca_{i}\right]}^{4}} \end{array}$$

where, *K*_*synt*_ is the calcium sensitivity, ${\bar{V}}_{synt}$ is the maximal velocity for L-DOPA synthesis, and [*Ca*_*i*_] is the intracellular calcium concentration.

The flux of synthesised L-DOPA, *J*_*synt*_, whose velocity is the function of intracellular calcium concentration and L-DOPA synthesis is regulated by the substrate (TYR) itself, extracellular DA (via autoreceptors) and intracellular DA concentrations, is given by,


(44) $$\begin{array}{l} J_{synt}=\frac{V_{synt}}{1+\frac{K_{TYR}}{\left[TYR\right]}*\left(1+\frac{\left[DA_{c}\right]}{K_{i,cda}}+\frac{\left[DA_{e}\right]}{K_{i,eda}}\right)} \end{array}$$

where, *V*_*synt*_ is the velocity of synthesising L-DOPA, [*TYR*] is the tyrosine concentration in terminal bouton, *K*_*TYR*_ is the tyrosine concentration at which half-maximal velocity was attained, *K*_*i,cda*_ is the inhibition constant on *K*_*TYR*_ due to cytosolic DA concentration, *K*_*i,eda*_ is the inhibition constant on *K*_*TYR*_ due to extracellular DA concentration, [*DA*_*c*_] is the cytoplasmic DA concentration, and [*DA*_*e*_] is the extracellular DA concentration. A detailed description of the SNc terminal is provided in section Biochemical Model of SNc Terminal of the Supplementary Material.

*Extracellular Dopamine:* The three major mechanisms that determine the dynamics of extracellular DA ([*DA*_*e*_]) in the extracellular compartment (ECS) given by,


(45) $$\begin{array}{l} \frac{d\left(\left[DA_{e}\right]\right)}{dt}=J_{rel}-J_{DAT}-J_{eda}^{o} \end{array}$$

(46) $$\begin{array}{l} \delta_{V}^{SNc}=F\left(DA_{e}\right) \end{array}$$

where, *J*_*rel*_ represents the flux of calcium-dependent DA release from the DA terminal, *J*_*DAT*_ represents the unidirectional flux of DA translocated from the ECS into the intracellular compartment (cytosol) via DA plasma membrane transporter (DAT), $J_{eda}^{o}$ represents the outward flux of DA degradation, which clears DA from ECS, and $\delta_{V}^{SNc}$ is the SNc-derived value difference. A detailed description of the SNc terminal is provided in section Biophysical Model of SNc of the Supplementary Material.

The cortical input to the striatum is modulated by the $\delta_{V}^{SNc}$ derived from the network of SNc neurons. When $\delta_{V}^{SNc}$ is high, the direct pathway will be selected, else the indirect pathway is selected.

### Time Scales of Various Loops

The time scales of various loops in the model are as given in Table 1. The STN-GPe loop runs with a timestep (dt) of 0.02 ms. Once the STN-GPe loop runs for 2,500 iterations, one timestep of the cortico-BG loop is run. Simultaneously, the SNc-STR loop which provides the modulatory signal for the selection of the Go-NoGo pathway in the striatum is run. For each timestep of the cortico-BG loop, STN-GPe and SNc-STR loops run 2,500 and 2,000 iterations, respectively. The total simulation time for the arm reaching task (cortico-BG loop) is 5 s and if the arm doesn't reach the target in the stipulated timeframe of 5 s, the trial is considered non-reachable. At the spatial level, the details of different loops are given in Figure 2.

**Table 1:** Timescales of various loops in the model.

	**Loops**	**Timescales**		
		**Timestep, dt (ms)**	**Total iterations**	**Total (ms)**
1	STN-GPe loop	0.02 ms	2,500	50 ms
2	SNc-Striatum loop	0.025 ms	2,000	50 ms
3	Cortico-BG loop	50 ms	100	5 s

#### Simulating Parkinsonian Conditions

To simulate the Parkinsonian condition in the present model, the number of neurons in the SNc population (network) was reduced. In order to kill the SNc neuron, we clamped their membrane voltage (*V*_*SNc*_) to resting membrane voltage (−80 *mV*). As the number of SNc neurons die the total amount of dopamine (*DA*_*e*_) that is made available to the striatum decreases. This influences the selection of the indirect pathway in the BG system over the direct pathway resulting in pathological conditions. In the present model, two types of PD conditions were simulated: in the first type, SNc cell loss affects striatum alone (PD1) and in the second type, SNc cell loss affects both striatum and STN (PD2).

In normal conditions, the SNc-derived value difference $\left(\delta_{V}^{SNc}\right)$ will be similar to the actual value difference computed (δ_*V*_). In case of PD1, the SNc-derived value difference $\left(\delta_{V}^{SNc}\right)$ will be lesser than the actual value difference computed (δ_*V*_). In the case of PD2, along with $\delta_{V}^{SNc}<\delta_{V}$, $\delta_{V}^{SNc}$ impacts the STN lateral connections, thereby influencing the complexity of the STN-GPe subsystem. The STN-GPe subsystem is an integral component of the indirect pathway and is believed to play a major role in exploratory behaviour (Sridharan et al., 2006; Chakravarthy and Balasubramani, 2014).

In normal condition:


(47) $$\begin{array}{l} \;\delta_{V}^{SNc}=F\left(DA_{e}\right)\\ \;DA_{e}=\mathrm{SNc}\left(I_{gaba},\;P_{SNc}\right);\;P_{SNc}=100\%\\ I_{gaba}=F\left(\delta_{V}\right)\\ \;\varepsilon_{s}=F\left(\delta_{V}^{SNc}\right) \end{array}$$

In PD1 condition:


(48) $$\begin{array}{l} \;\delta_{V}^{SNc}=F\left(DA_{e}\right)\\ \;DA_{e}=\mathrm{SNc}\left(I_{gaba},\;P_{SNc}\right);\;P_{SNc}<100\%\\ I_{gaba}=F\left(\delta_{V}\right)\\ \;\varepsilon_{s}=F\left(\delta_{V}\right) \end{array}$$

In PD2 condition:


(49) $$\begin{array}{l} \;\delta_{V}^{SNc}=F\left(DA_{e}\right)\\ \;DA_{e}=\mathrm{SNc}\left(I_{gaba},\;P_{SNc}\right);\;P_{SNc}<100\%\\ I_{gaba}=F\left(\delta_{V}\right)\\ \;\varepsilon_{s}=F\left(\delta_{V}^{SNc}\right) \end{array}$$

where, $\delta_{V}^{SNc}$ is the SNc-derived value difference, δ_*V*_ is the value difference computed, *DA*_*e*_ is the extracellular dopamine, *I*_*gaba*_ is the inhibitory GABAergic current from the striatum, *P*_*SNc*_ is the percentage of SNc neurons, and ε_*s*_ is the lateral connection strength in the STN network.

#### Levodopa Medication

When a drug is administered to a patient, the medication action is broadly classified into two major branches: pharmacokinetics (what the body does to the drug) and pharmacodynamics (what the drug does to the body) (Shanbhag and Shenoy, 2020).

##### Pharmacokinetics

**Figure 3:**
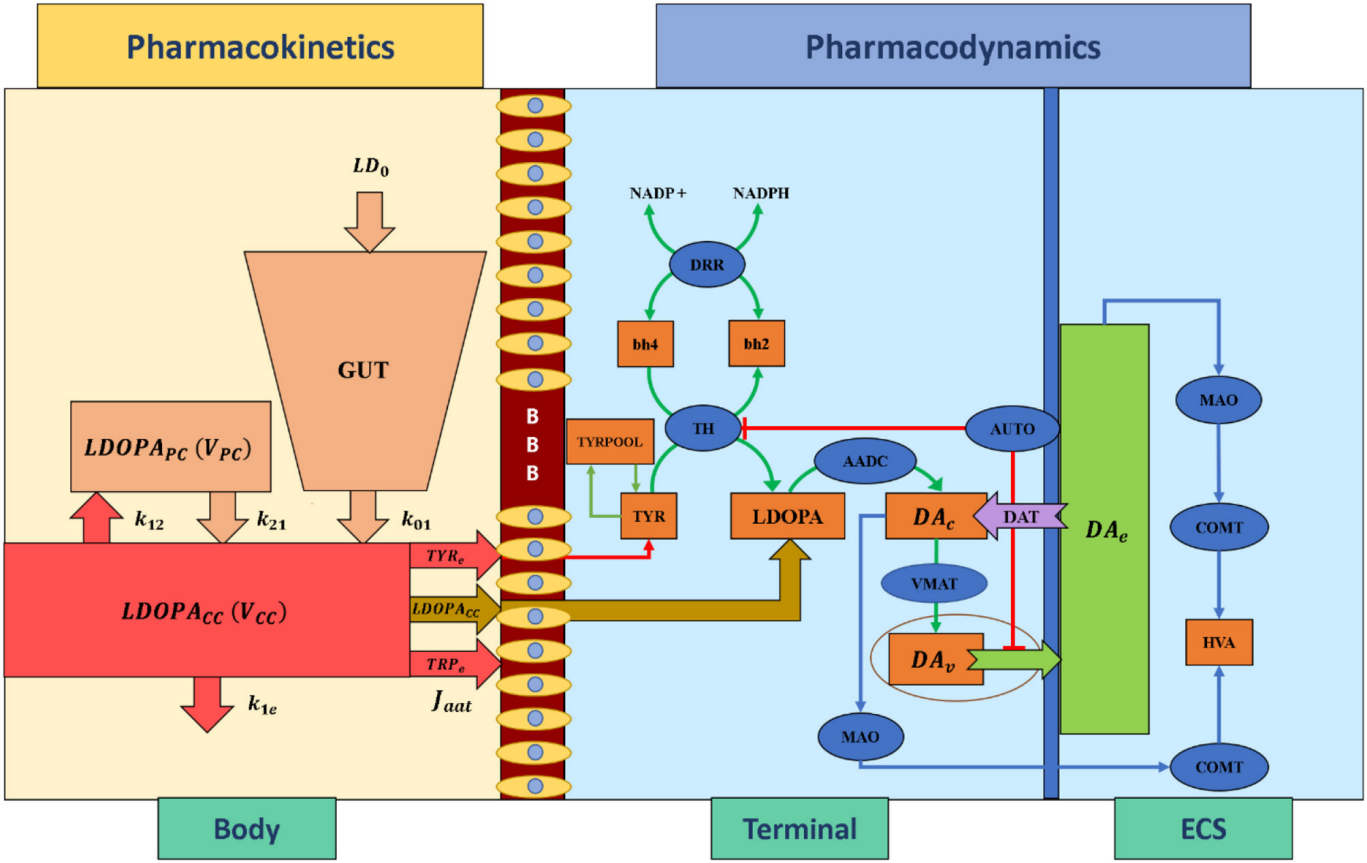
Schematic diagram of pharmacokinetics and pharmacodynamics of levodopa medication. BBB, blood-brain barrier; LDOPA, intracellular levodopa; *LDOPA*_*CC*_, levodopa in the central compartment; *LDOPA*_*PC*_, levodopa in the peripheral compartment; *V*_*CC*_, the volume of the central compartment; *V*_*PC*_, the volume of the peripheral compartment; *TYR*_*e*_, extracellular tyrosine; *TRP*_*e*_, extracellular tryptophan; *k*_21_, rate constant from peripheral to central compartments, *k*_12_, rate constant from central to peripheral compartments, *k*_1*e*_, total clearance rate constant from the central compartment, *k*_01_, the infusion rate of *LD*_0_ into the central compartment, *LD*_0_, levodopa dose; *J*_*aat*_, the flux of exogenous L-DOPA transported into the terminal through aromatic L-amino acid transporter; ECS, extracellular space; *DA*_*c*_, cytosolic dopamine; *DA*_*v*_, vesicular dopamine; *DA*_*e*_, extracellular dopamine; TYR, tyrosine; TRYPOOL, tyrosine pool; HVA, homovanillic acid; bh2, dihydrobiopterin; bh4, tetrahydrobiopterin; NADP+, nicotinamide adenine dinucleotide phosphate; NADPH, nicotinamide adenine dinucleotide phosphate hydrogen; TH, tyrosine hydroxylase; DDR, dihydropteridine reductase; AADC, aromatic amino acid decarboxylase; VMAT, vesicular monoamine transporter; DAT, dopamine transporter; AUTO, dopamine autoreceptors; MAO, monoamine oxidase; COMT, catecholamine methyltransferase.

Pharmacokinetics deals with the absorption, distribution, metabolism, and excretion of drugs. In the present study, we have adapted a two-compartment pharmacokinetic model of levodopa (L-DOPA) (Baston et al., 2016), which consists of central and peripheral compartments (Figure 3). Orally consumed L-DOPA is absorbed in the intestine and reaches the bloodstream. The bloodstream carries the drug all over the body. Proteins break down L-DOPA and around three-fourth of the drug is deactivated before it even reaches the brain. The central compartment where L-DOPA is administered and plasma L-DOPA concentration was measured which is defined as,


(50) $$\begin{array}{r} V_{CC}\frac{d\left[LDOPA_{CC}\right]}{dt}=k_{01}LD_{0}+k_{21}\left[LDOPA_{PC}\right]\\ -\left(k_{12}+k_{1e}\right)\left[LDOPA_{CC}\right] \end{array}$$

where, *V*_*CC*_ is the volume of the central compartment, [*LDOPA*_*CC*_] is the L-DOPA concentration in the central compartment, *LD*_0_ is the L-DOPA dose (in milligramme), [*LDOPA*_*PC*_] is the L-DOPA concentration in peripheral compartment, *k*_01_ is the infusion rate of *LD*_0_ into the central compartment, *k*_21_ is the rate constant from peripheral to central compartments, *k*_12_ is the rate constant from central to peripheral compartments, and *k*_1*e*_ is the total clearance rate constant from the central compartment.

The interaction between plasma L-DOPA and other body fluids, which occurs in the peripheral compartment, is defined as,


(51) $$\begin{array}{l} V_{PC}\frac{d\left[LDOPA_{PC}\right]}{dt}=k_{12}\left[LDOPA_{CC}\right]-k_{21}\left[LDOPA_{PC}\right] \end{array}$$

where, *V*_*PC*_ is the volume of the peripheral compartment, [*LDOPA*_*CC*_] is the L-DOPA concentration in the central compartment, [*LDOPA*_*PC*_] is the L-DOPA concentration in peripheral compartment, *k*_21_ is the rate constant from peripheral to central compartments, and *k*_12_ is the rate constant from central to peripheral compartments.

##### Pharmacodynamics

Pharmacodynamics deals with molecular, biochemical, and physiological effects of drugs, including drug mechanism of action, receptor binding (including receptor sensitivity), postsynaptic receptor effects, and chemical interactions. In the present study, we have adapted a three-compartment dopaminergic terminal model (Reed et al., 2012) which consists of extracellular, vesicular, and cytoplasmic compartments.

When L-DOPA medication is administered, the flux of exogenous L-DOPA ([*LDOPA*_*CC*_]) transported into the terminal through aromatic L-amino acid transporter (AAT) while competing with other aromatic amino acids [such as tyrosine (TYR) and tryptophan (TRP)] (Reed et al., 2012) is given by,


(52) $$\begin{array}{l} J_{aat}={\bar{V}}_{aat}\\ \;*\frac{\left[LDOPA_{CC}\right]}{\left(K_{ldopa_{e}}*\left(1+\left(\frac{\left[TYR_{e}\right]}{K_{tyr_{e}}}\right)+\left(\frac{\left[TRP_{e}\right]}{K_{trp_{e}}}\right)\right)+\left[LDOPA_{CC}\right]\right)} \end{array}$$

where, *K*_*ldopa*_*e*__ is the extracellular L-DOPA concentration at which half-maximal velocity was attained, ${\bar{V}}_{aat}$ is the maximal velocity with which extracellular L-DOPA was transported into the cytosol, [*LDOPA*_*CC*_] is the extracellular (central compartment) L-DOPA concentration, [*TYR*_*e*_] is the extracellular TYR concentration, [*TRP*_*e*_] is the extracellular TRP concentration, *K*_*tyr*_*e*__ is the affinity constant for [*TYR*_*e*_], *K*_*trp*_*e*__ is the affinity constant for [*TRP*_*e*_ ].

The L-DOPA concentration ([*LDOPA*]) dynamics inside the terminal is given by,


(53) $$\begin{array}{l} \frac{d\left(\left[LDOPA\right]\right)}{dt}=J_{aat}-J_{ldopa}+J_{synt} \end{array}$$

where, *J*_*aat*_ represents the flux of exogenous L-DOPA ([*LDOPA*_*CC*_]) transported into the cytosol, *J*_*ldopa*_ represents the conversion flux of exogenous L-DOPA ([*LDOPA*_*CC*_]) into dopamine, and *J*_*synt*_ represents the flux of synthesised L-DOPA from tyrosine. A detailed description of the dopaminergic terminal is provided in section Biochemical model of dopaminergic terminal of the Supplementary Material.

#### Timescales in the Model

Reaching movements, like several other behavioural events, involve dynamics at multiple timescales: the neuronal activity which is generally in milliseconds, and the actual movement which unfolds over the order of seconds. In the present model, the outer (sensory-motor) loop is assumed to run slightly slower than the inner (cortico-basal ganglia) and central (nigrostriatal) loops. As the dynamics of the STN–GPe loop in the indirect pathway needs some time to settle, we run this loop for 2, 500 iterations (*dt* = 0.02 *ms*), before sending the output to the MC (MC runs for 100 iterations with *dt* = 50 *ms*). Thus, a single update of the MC activity happens after every 50 *ms* during which the BG dynamics run. Similarly, since the dynamics of the SNc neuron needs some time to settle, we run the SNc neuron for 2, 000 iterations (*dt* = 0.025 *ms*), before sending the output to the BG. Thus, a single update of the MC activity happens after every 50 *ms* during which the SNc dynamics run. All the results presented are at the timescale of the MC.

In the present model, the SNc neurons run in milliseconds timescale whereas the pharmacokinetic-pharmacodynamic model of L-DOPA medication runs in hourly timescale. In order to show the drug effect, we sample various points across the L-DOPA medication curve (Supplementary Figure 7.1) and simulated the MCBG model for the arm reaching task for each sampled point.

## Results

**Figure 4:**
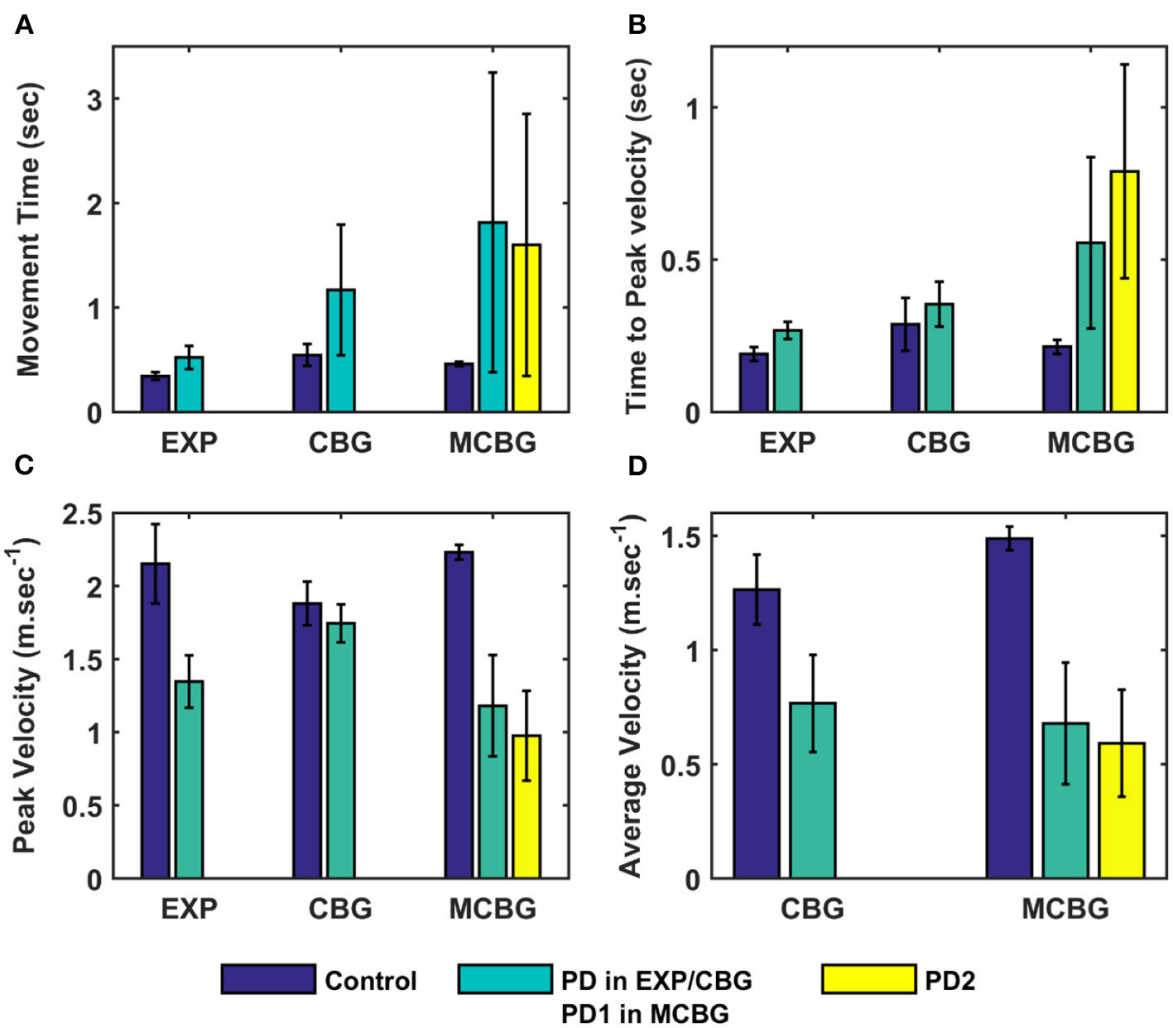
Comparison of performance of the proposed model (during the testing phase) with CBG model (Muralidharan et al., 2018) and experimental data adapted from (Majsak et al., 1998). **(A)** Movement time, **(B)** Time-to-peak velocity, and **(C)** Peak velocity, **(D)** Average velocity. EXP, experiment; CBG, cortico-basal ganglia model; MCBG, multiscale cortico-basal ganglia model; PD1, only striatum affected; PD2, both striatum and subthalamic nucleus affected; sec, second; m/sec, meter per second.

**Figure 5:**
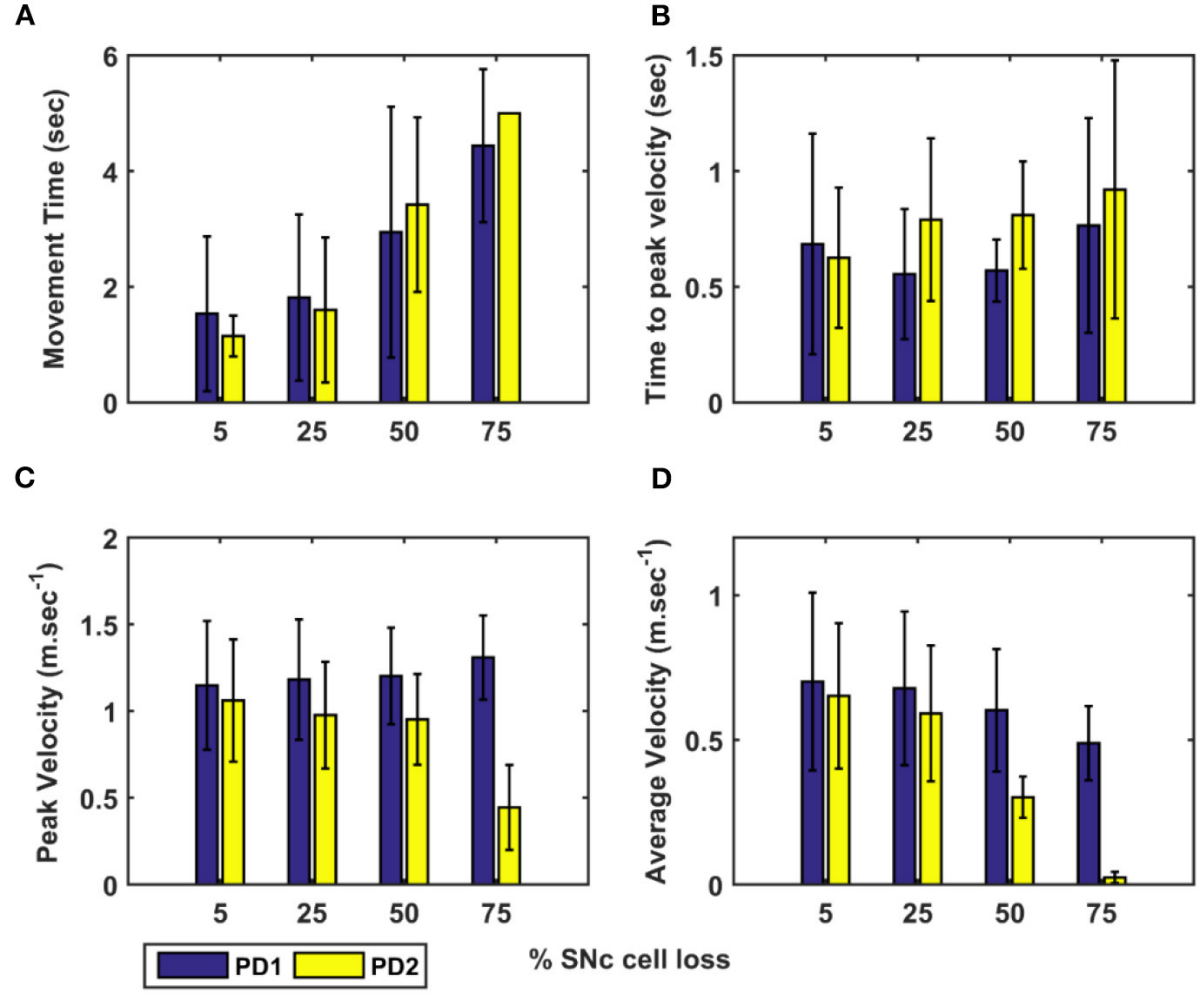
Performance of arm reaching for various PD conditions across different percentages of SNc cell loss. **(A)** Movement time, **(B)** Time-to-peak velocity, **(C)** Peak velocity, and **(D)** Average velocity. SNc, substantia nigra pars compacta; PD1, SNc cell loss affecting striatum only; PD2, SNc cell loss affecting both striatum and subthalamic nucleus; sec, second; m.sec^−1^, metre per second.

**Figure 6:**
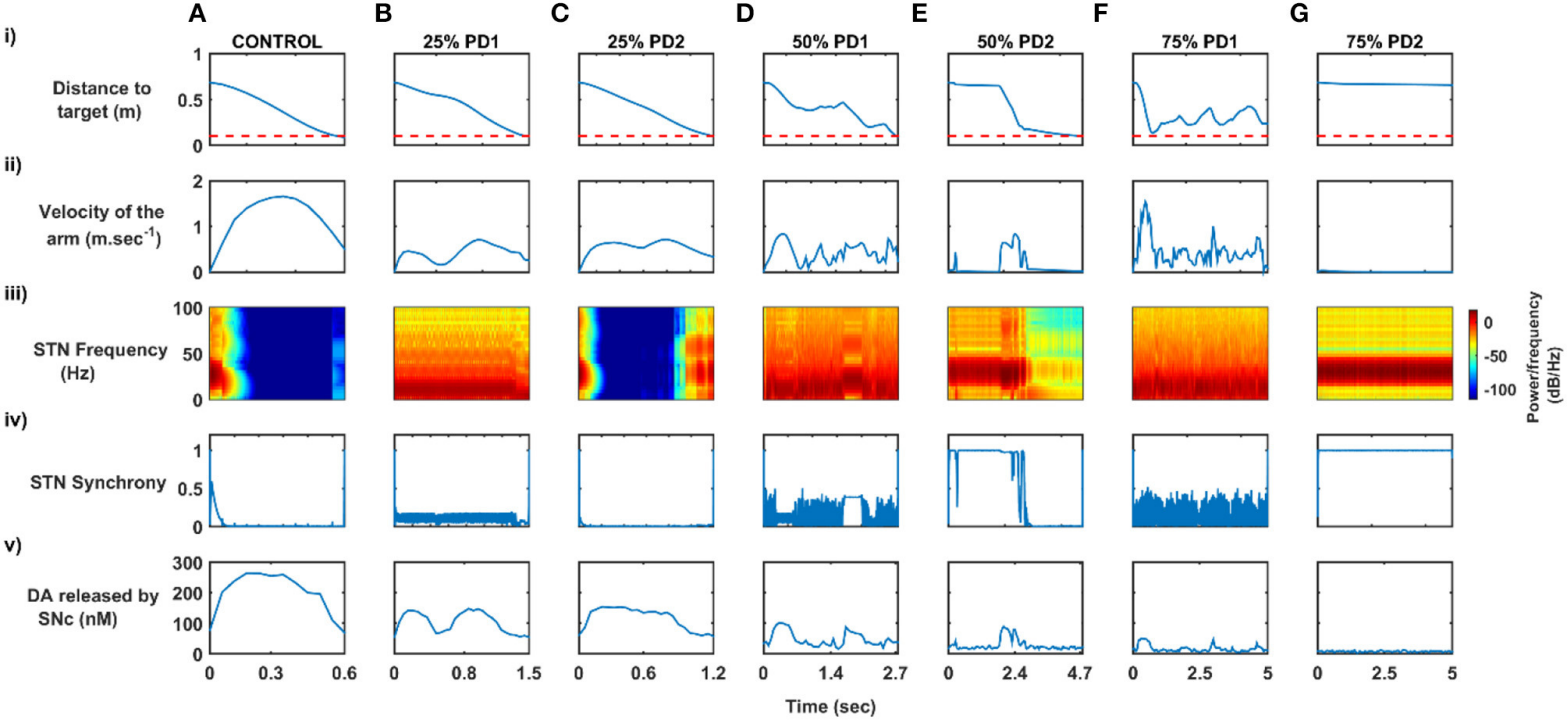
Differential dopaminergic axonal degeneration manifesting in terms of various PD motor symptoms. **(A)** Control, **(B)** 25% PD1, **(C)** 25% PD2, **(D)** 50% PD1, **(E)** 50% PD2, **(F)** 75% PD1, and **(G)** 75% PD2, (i) Distance to target, (ii) Velocity of the arm, (iii) Spectrogram of STN population, (iv) Synchrony in STN population, (v) Dopamine released by SNc extracellularly. SNc, substantia nigra pars compacta; STN, subthalamic nucleus; STR, striatum; DA, dopamine; PD, Parkinson's disease; sec, second; m/sec, meter per second; Hz, hertz; nM, nanomolar.

**Figure 7:**
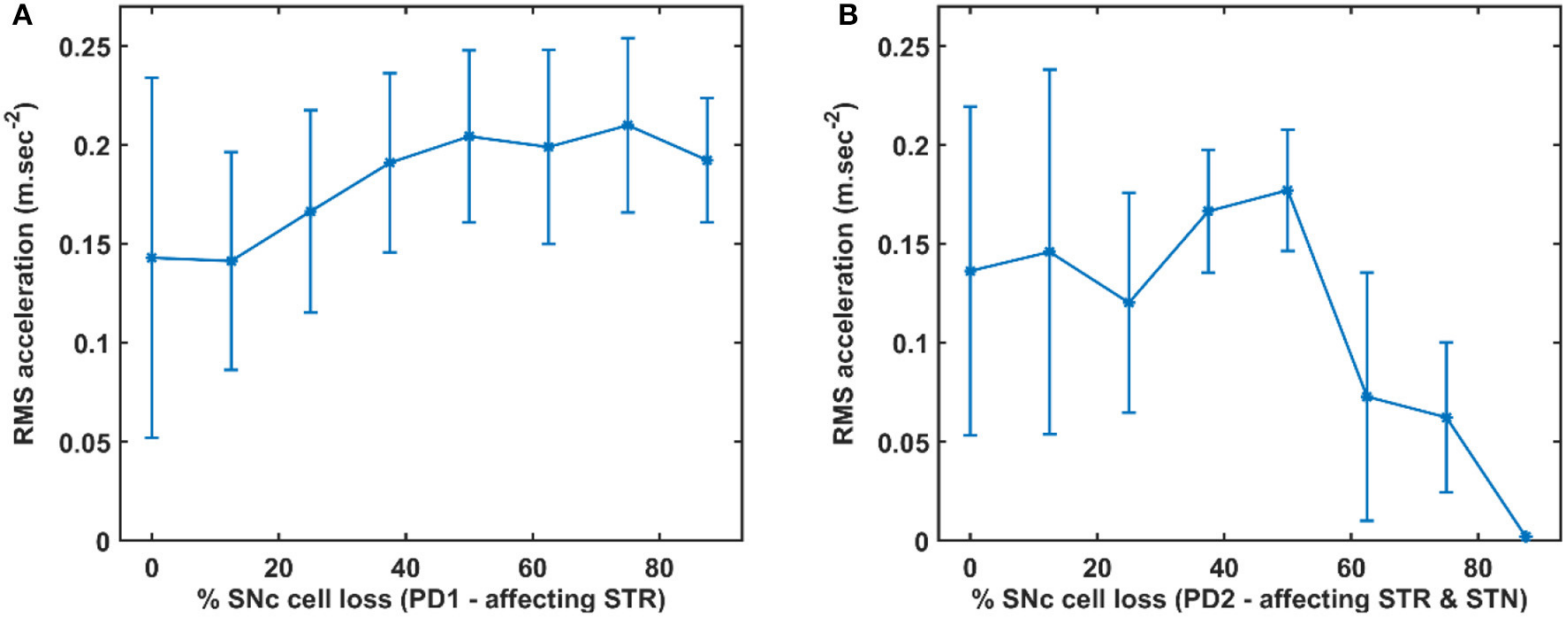
RMS acceleration with respect to the percentage loss of SNc cells. **(A)** RMS acceleration when SNc cell loss affecting STR. **(B)** RMS acceleration when SNc cell loss affecting STR & STN. SNc, substantia nigra pars compacta; STN, subthalamic nucleus; STR, striatum; PD1, SNc cell loss affecting STR; PD2, SNc cell loss affecting STR & STN; RMS, root mean squared; m/sec^2^, metre per second squared.

**Figure 8:**
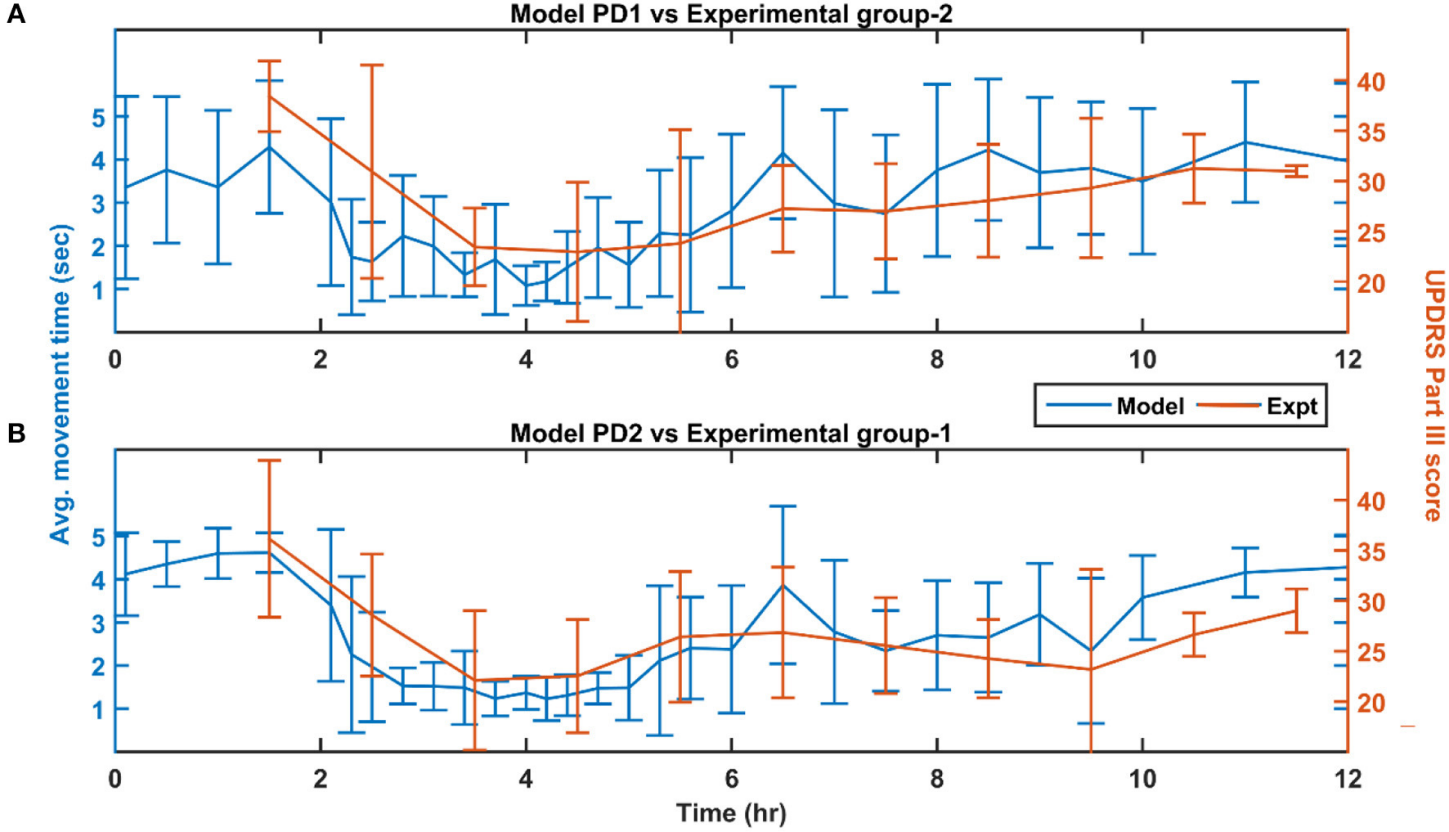
Performance of the model (150 mg L-DOPA and 62% SNc cell loss) compared with experimental study (~140 mg L-DOPA) (Nomoto et al., 2018) for various PD conditions. **(A)** Movement time of PD1 MCBG model was compared with UPDRS Part III score of experimental group-2 after L-DOPA administration. **(B)** Movement time of PD2 MCBG model was compared with UPDRS Part III score of experimental group-1 after L-DOPA administration. MCBG, multiscale cortico-basal ganglia model; L-DOPA, levodopa; PD, Parkinson's disease; PD1, when SNc cell loss affecting STR alone; PD2, when SNc cell loss affecting both STR & STN; SNc, substantia nigra pars compacta; STR, striatum; STN, subthalamic nucleus; UPDRS, Unified Parkinson's disease rating scale; Expt, experiment; mg, milligramme; sec, second; hr, hour.

**Figure 9:**
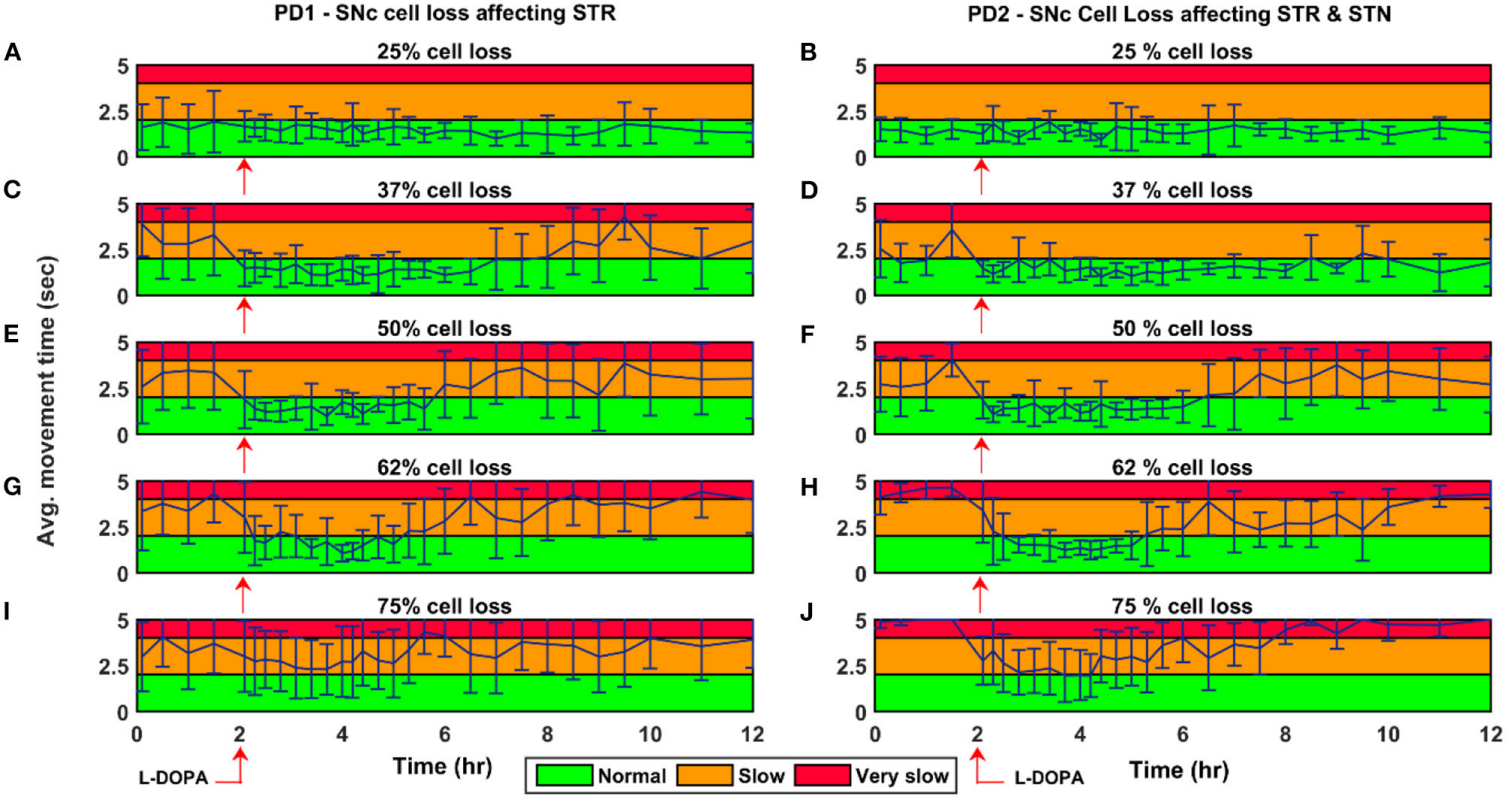
Average time to reach the target for 150 mg L-DOPA medication for various PD conditions. Average movement time for SNc cell loss of 25% **(A,B)**, 37% **(C,D)**, 50% **(E,F)**, 62% **(G,H)**, and 75% **(I,J)** when SNc cell loss affecting STR (PD1) and STR & STN (PD2) during L-DOPA medication (administrated at the second hour, indicated by red arrow). The performance of the model during L-DOPA medication is categorised into three regions based on movement time. Green region—when the arm reaches the target within 2 s; Yellow region—when the arm reaches the target between 2 and 4 s; Red region—when the arm reaches the target beyond 4 s. PD1, SNc cell loss affecting STR; PD2, SNc cell loss affecting STR & STN; SNc, substantia nigra pars compacta; STN, subthalamic nucleus; STR, striatum; L-DOPA, levodopa; sec, second; hr, hour.

**Figure 10:**
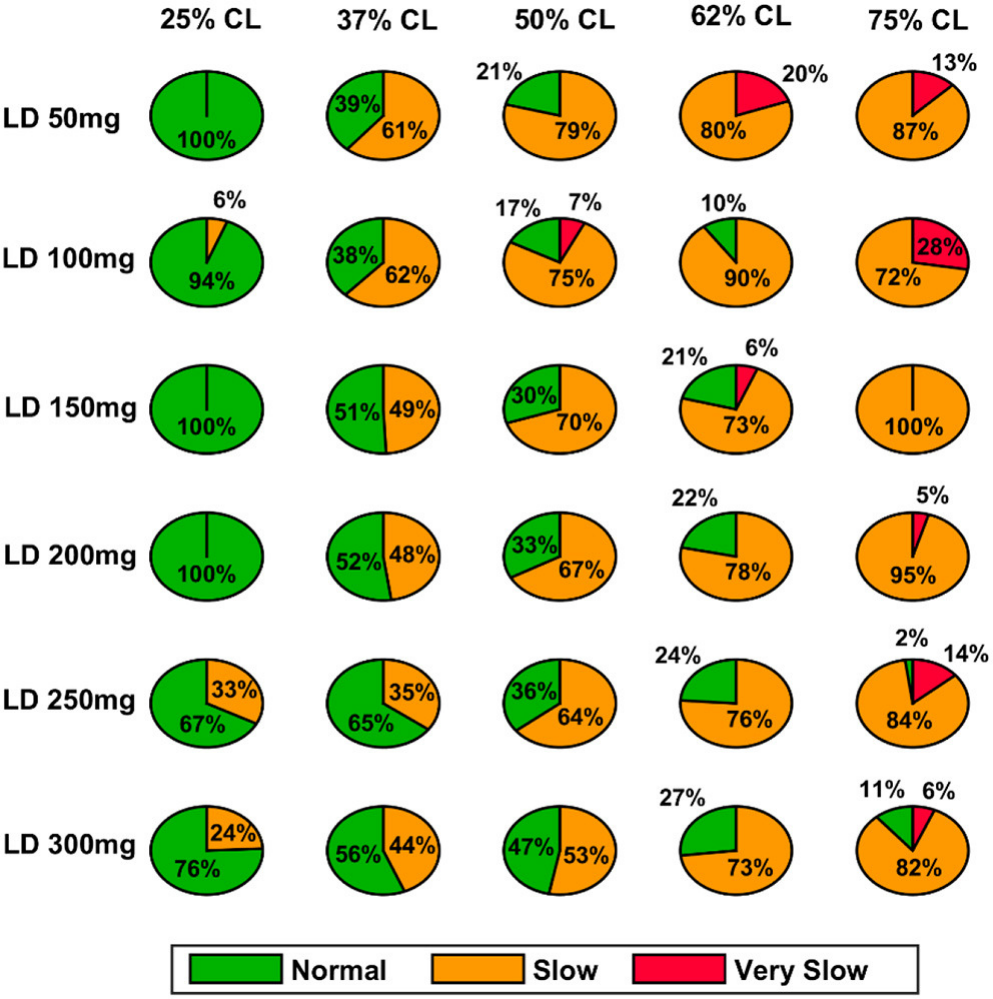
Model performance for different L-DOPA dosage across various percentage SNc cell loss where SNc cell loss affects STR–PD1. CL, cell loss; LD or L-DOPA, levodopa; SNc, substantia nigra pars compacta; STR, striatum.

**Figure 11:**
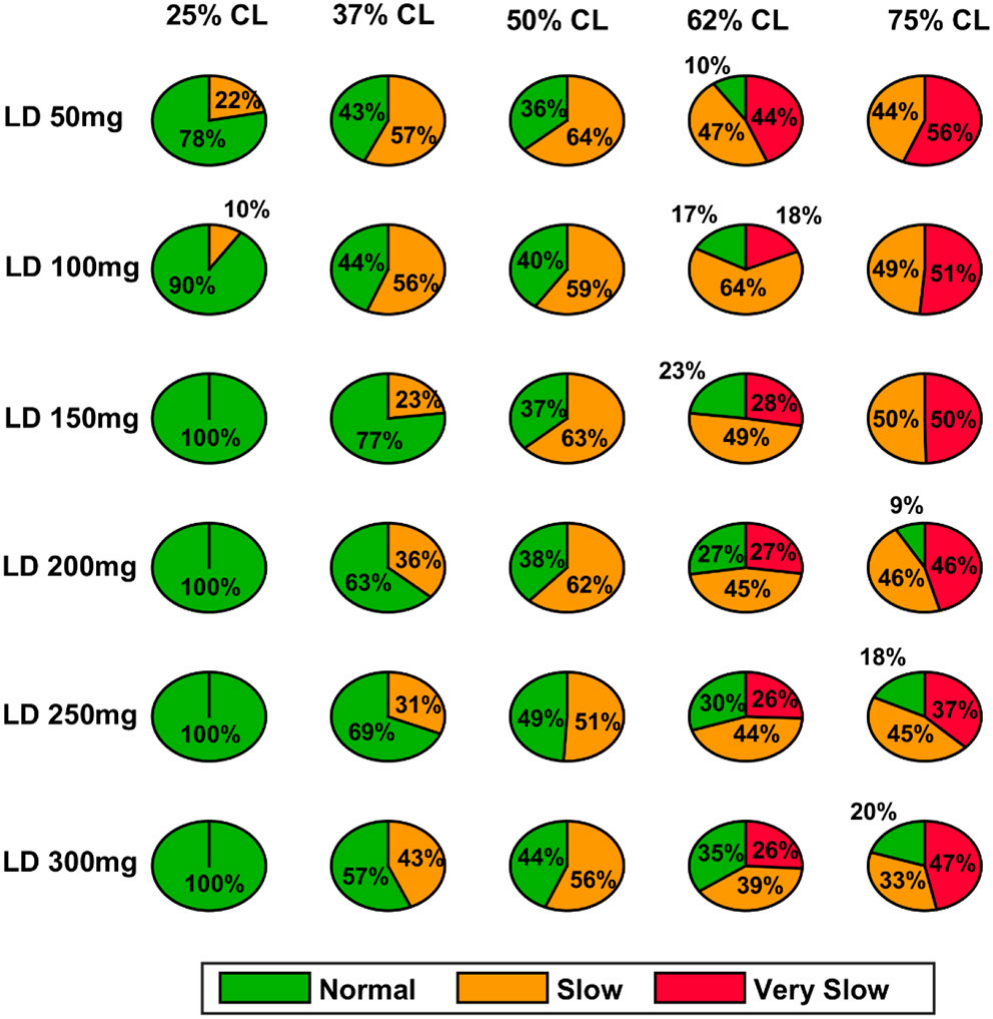
Model performance for different L-DOPA dosage across various percentage SNc cell loss where SNc cell loss affects STR & STN–PD2. CL, cell loss; LD or L-DOPA, levodopa; SNc, substantia nigra pars compacta; STR, striatum.

**Figure 12:**
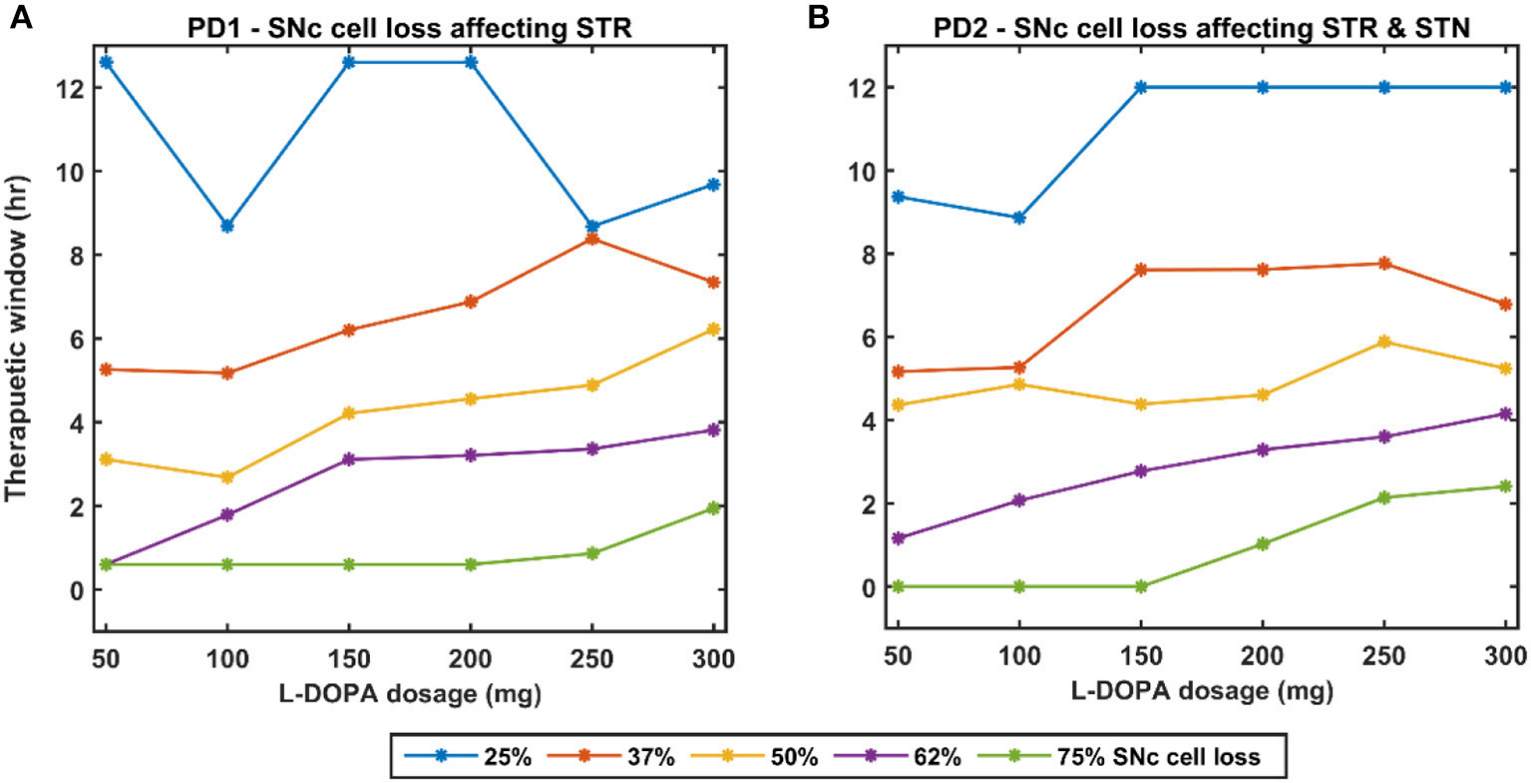
Effect of L-DOPA dosage on the therapeutic window for various PD conditions. **(A)** Therapeutic window across different L-DOPA dosage for various percentages of SNc cell loss when SNc cell loss affecting STR. **(B)** Therapeutic window across different L-DOPA dosage for various percentages of SNc cell loss when SNc cell loss affecting STR & STN. PD1, SNc cell loss affecting STR; PD2, SNc cell loss affecting STR & STN; SNc, substantia nigra pars compacta; STN, subthalamic nucleus; STR, striatum; L-DOPA, levodopa; mg, milligramme; hr, hour.

Here, we showcase the performance of the model by simulating the PD condition and read out their effects on behavioural outcomes (Figures 4, 5). Furthermore, demonstrates the effect of differential dopaminergic axonal loss manifest into some of the cardinal symptoms of PD (Figures 6, 7). Next, assessing the performance in terms of reaching time and verifying the effect of L-DOPA therapeutic intervention (Figures 8, 9). Finally, describing the model results which gave an indicator of how to optimise the drug dosage across the course of the disease progression (Figures 10–12).

### Testing Phase

The performance of the MCBG model was tested, the model results were compared to that of the CBG model (Muralidharan et al., 2018) and the experimental data (Majsak et al., 1998) for both control and PD conditions. In the MCBG model, PD conditions simulated were subdivided into two categories: in PD1, the SNc cell loss impacts only striatum whereas in PD2, the SNc cell loss impacts both striatum and STN. The MCBG and CBG models were tested and the performance was evaluated with respect to movement time, peak velocity, time-to-peak velocity, and average velocity along with the experimental results. In the control case, the MCBG model reaches the target in 0.46 ± 0.02 *s* compared to the CBG model and the experimental subject which reaches the target in 0.56 ± 0.1 *s* and 0.3432 ± 0.04 *s*, respectively (Figure 4A, dark blue bar). The MCBG model obtained a peak velocity of 2.23 ± 0.05 *ms*^−1^ compared to the CBG model and experimental subject which obtained peak velocity of 1.88 ± 0.15 *ms*^−1^ and 2.15 ± 0.27 *ms*^−1^, respectively, during the arm reaching toward the target in case of control (Figure 4C, dark blue bar). The time taken to reach the peak velocity in the case of control was 0.21 ± 0.02 *s* for the MCBG model, 0.29 ± 0.09 *s* for the CBG model, and 0.19 ± 0.02 *s* for the experimental subject (Figure 4B, dark blue bar). Finally, the average velocities for MCBG and CBG models were found to be 1.49 ± 0.05 *ms*^−1^ and 1.26 ± 0.15 *ms*^−1^, respectively, in the case of control (Figure 4D, dark blue bar).

In the case of PD, the experimental subject recorded an average movement time of 0.52 ± 0.63 *s*, respectively (Figure 4A, cyan bar), while the CBG model reaches the target in 1.17 ± 0.63 *s* (Figure 4A, cyan bar) whereas the MCBG model took 1.88 ± 1.42 *s* and 1.6 ± 1.35 *s* for PD1 (Figure 4A, cyan bar) and PD2 (Figure 4A, yellow bar), respectively. The experimental subject recorded a peak velocity of 1.35± 0.18 *ms*^−1^ (Figure 4C, cyan bar) compared to the CBG model which obtained a peak velocity of 1.74 ± 0.13 *ms*^−1^ (Figure 4C, cyan bar) whereas the MCBG model obtained peak velocities of 1.18 ± 0.35 *ms*^−1^ (Figure 4C, cyan bar) and 0.98 ± 0.31 *ms*^−1^ (Figure 4C, yellow bar), respectively, during the arm trajectory toward the target. The time taken to reach the peak velocity in the PD case was 0.27 ± 0.03 *s* for the experimental subject (Figure 4B, cyan bar), 0.35 ± 0.07 *s* for CBG model (Figure 4B, cyan bar) and 0.56 ± 0.28 *s*, and 0.79 ± 0.35 *s* in PD1 (Figure 4B, cyan bar) and PD2 (Figure 4B, yellow bar) cases, respectively, for MCBG model. Finally, the average velocity for the CBG model was found to be 0.77 ± 0.21 *ms*^−1^ in PD (Figure 4D, cyan bar), and the average velocities for the MCBG model were found to be 0.68 ± 0.27 *ms*^−1^ and 0.59 ± 0.23*ms*^−1^ in PD1 (Figure 4D, cyan bar) and PD2 (Figure 4D, yellow bar), respectively.

### Simulating Parkinsonian Conditions

To simulate PD conditions in the model, SNc cells were killed and their effects on basal ganglia were considered in two aspects. In the first scenario, only the striatum is affected by SNc cell loss (PD1—cell loss affecting nigrostriatal pathway only) and in the second scenario, both striatum and STN are affected by SNc cell loss (PD2—cell loss affecting both nigrostriatal and nigrosubthalamic pathways).

#### Effect of SNc Cell Loss on MCBG Behavioural Outcome

To assess the performance metrics with respect to dopaminergic cell loss affecting striatum and both striatum and STN, a comparison study was done with respect to the movement time, peak velocity, time required to peak velocity, and average velocity (Figure 5). In both cases (PD1 and PD2), the time required to reach the target (Figure 5A) and time-to-reach the peak velocity (Figure 5B) increases with an increase in SNc cell loss. In the PD1 case, the peak velocity increases with an increase in SNc cell loss when compared to the PD2 case where the peak velocity decreases with an increase in SNc cell loss (Figure 5C). The reason behind this discrepancy in both cases will be explored in the next sections where one leads to tremor-like behaviour and the other leads to rigidity-like behaviour. In both cases, the average velocity across the trajectory decreases with an increase in SNc cell loss (Figure 5D).

#### Differential Dopaminergic Axonal Degeneration Manifests Into Various PD Motor Symptoms

Both the PD scenarios (PD1 and PD2) simulated in the model can be attributed to differential degeneration of dopaminergic projections to various targets in the basal ganglia, and how degeneration manifests into various motor symptoms of PD. In the control case, the arm reaches the target in 0.55 *s* (Figure 6Ai) with the peak velocity of 1.91 *ms*^−1^ (Figure 6Aii). The population activity of STN exhibits desynchronous activity during the arm movement which is indicated in the STN spectrogram (Figure 6Aiii) and synchrony (average value = 0.03) (Figure 6Aiv) (synchrony measure is described in section Network analysis of the Supplementary Material). Dopamine released by SNc neurons in the striatum during the arm reaching peaked at ~ 264 *nM* which was in the range of 150–400 nM (Schultz, 1998; Figure 6Av).

In 25% PD1, the arm reaches the target in 1.5 *s* (Figure 6Bi) with a reduced peak velocity of 0.71 *ms*^−1^, exhibiting bradykinesia-like behaviour in the arm (Figure 6Bii). Population activity of STN exhibits greater synchrony compared to control case during the arm movement which is also indicated in STN spectrogram (Figure 6Biii) and synchrony with an average value of 0.11 (Figure 6Biv). Dopamine released by SNc neurons in the striatum during the arm reaching peaked at ~ 148 *nM* which was lesser than in the control case (Figure 6Bv).

In 25% PD2, the arm reaches the target in 1.2 *s* (Figure 6Ci) with the peak velocity of 0.71 *ms*^−1^, exhibiting bradykinesia-like behaviour in the arm (Figure 6Cii). Population activity of STN exhibits desynchronous activity, same as control case during the arm movement which is indicated in the STN spectrogram (Figure 6Ciii) and synchrony with an average value of > 0.01 (Figure 6Civ). Dopamine released by SNc neurons in the striatum during the arm reaching peaked at ~ 154 *nM* which was lesser than the control case (Figure 6Cv).

In 50% PD1, the arm reaches the target in 2.7 *s* (Figure 6Di) with the peak velocity of 0.84 *ms*^−1^ showing tremor-like behaviour in the arm (Figure 6Dii). Population activity of STN exhibits low synchronous activity during the arm movement which indicates in STN spectrogram (Figure 6Diii) and synchrony with an average value of 0.17 (Figure 6Div). Dopamine released by SNc neurons in the striatum during the arm reaching peaked at ~ 101 *nM* which was lesser than the control case (Figure 6Dv). In 50% PD2, the arm reaches the target in 4.7 *s* (Figure 6Ei) with the peak velocity of 0.84 *ms*^−1^ as a result of cogwheel-like behaviour in the arm (Figure 6Eii). The population activity of STN exhibits high synchronous activity during the arm movement which indicates in the STN spectrogram (Figure 6Eiii) and synchrony with an average value of 0.55 (Figure 6Eiv). Dopamine released by SNc neurons in the striatum during the arm reaching peaked at ~ 90 *nM* which was lesser than the control case (Figure 6Ev).

In 75% PD1, the arm did not reach the target within 5 *s* (Figure 6Fi) with the peak velocity of 1.54 *ms*^−1^ displaying a tremor-like behaviour in the arm (Figure 6Fii). Population activity of STN exhibits low synchronous activity during the arm movement which indicates in STN spectrogram with increased power in 5 − 25 *Hz* region (Figure 6Fiii) and synchrony with an average value of 0.15 (Figure 6Fiv). Dopamine released by SNc neurons in the striatum during the arm reaching peaked at ~ 51 *nM* which was lesser than the control case (Figure 6Fv). In 75% PD2, the arm did not reach the target within 5 *s* (Figure 6Gi) with zero peak velocity as a result of the rigidity-like state of the arm (Figure 6Gii). Population activity of STN exhibits high synchronous activity during the arm movement which is indicated in STN spectrogram with increased power in 15−50 *Hz* region (Figure 6Giii) and synchrony with an average value of > 0.99 (Figure 6Giv). Dopamine released by SNc neurons in the striatum during the arm reaching peaked at ~ 13 *nM*, which was lesser than in the control case (Figure 6Gv).

#### Quantifying Tremor-Like and Rigidity-Like Motor Symptoms

To quantify between tremor-like and rigidity-like motor symptoms of PD, root mean square (RMS) acceleration was computed across movement trajectory for various PD conditions where RMS acceleration can be used as an indicator of random non-deterministic movements (Figure 7). In the PD1 scenario, the RMS acceleration increases with an increase in SNc cell loss which indicates irregular changes in the velocity of arm movement (Figure 7A). This irregular velocity profile in PD1 is a result of tremor-like motor behaviour. In the PD2 scenario, the RMS acceleration increases with an increase in SNc cell loss to 50%, and beyond 50% RMS acceleration decreases with an increase in SNc cell loss (Figure 7B). The tremor-like motor behaviour is indicated by the RMS acceleration increases until 50% SNc cell loss and from there on, we can see a sudden decrease, which marks the onset of rigidity.

### Effect of Levodopa Medication

In order to show the L-DOPA medication effect on the MCBG model, we simulated different scenarios where various L-DOPA dosages were administrated across various PD conditions and movement time was monitored.

#### Comparison of MCBG Model With Experimental Results

The L-DOPA therapeutic effect was monitored by recording the performance in terms of the average movement time across the time course of the dosage for the next 10 h. The performance of the model was also recorded 2 h prior to the administration of the drug. The MCBG model results were compared with experimental studies where PD patients were evaluated based on UPDRS Part III score (Nomoto et al., 2018; Figure 8). The experimental PD subjects were categorised into two groups based on the UPDRS part III score (motor evaluation) where the group 1 PD subjects have a mean UPDRS III score of 28 (13–51) and the group 2 PD have a mean UPDRS III score of 30.3 (22–41) (Nomoto et al., 2018). An average L-DOPA dosage of 141 mg was given to both the experimental groups. The MCBG model was simulated with 62% SNc cell loss and 150 mg of L-DOPA administered at the second hour of the simulation.

The PD1 MCBG model performance in terms of movement time (Figure 8A, blue curve) matched with experimental group 2 result in terms of UPDRS III score (Figure 8A, orange curve). Similarly, PD2 MCBG model performance in terms of movement time (Figure 8B, blue curve) matched with experimental group 1 result in terms of UPDRS III score (Figure 8B, orange curve).

#### Effect of L-DOPA Medication With Disease Progression

The effect of L-DOPA (150 mg) medication on the model performance was studied across different percentages (25, 37, 50, 62, and 75%) of SNc cell loss for both PD1 and PD2 scenarios. The L-DOPA medication was given at the second hour in the simulation. The simulated results show that as SNc cell loss increases, the model performance deteriorates, and also the therapeutic effect decreases as the disease progresses in both PD1 and PD2 scenarios (Figure 9). The maximum therapeutic effect of L-DOPA was seen for 50% and 62% SNc cell loss in both PD1 and PD2 scenarios (Figures 9E–H). In 75% SNc cell loss, the model performance was poor in case of PD1 when compared to PD2 (Figures 9I,J). The model performance was categorised into three regions based on the following criteria: If the arm reaches the target within 2 s, then that region was marked in green colour which indicates the normal movement. If the arm reaches the target between 2 and 4 s then that region was marked in yellow colour, indicating slow movement or *bradykinesia*. If the arm reaches the target beyond 4 s, then that region was marked in red colour which indicates very slow movement or *akinesia*. The simulated results show that as the SNc cell loss increases the movement time curve shift from green to the yellow region when medication was ON and the movement time curve shift from yellow to the red region when medication was OFF (Figure 9).

#### Effect of L-DOPA Dosage and SNc Cell Loss on Therapeutic Window

As discussed in the previous section, the model performance was categorised into three regions: green (normal movement), yellow (slow movement, bradykinesia), and red (very slow movement, akinesia). The therapeutic window is computed by taking the time difference between the points when the performance improved after taking medication and entered into the green shaded region until it started wearing off and crosses back to the yellow shaded region (where the effects of L-DOPA start wearing off).

In the case of 25%, SNc cell loss (PD1), as the L-DOPA dosage increases the therapeutic window (green region) decreases (Figure 10, first column). But at higher percentage loss of cells (37, 50, 62, and 75% SNc cell loss), as the L-DOPA dosage increases the therapeutic window (green region) increased (Figure 10). However, in the case of PD2 for all percentages of SNc cell loss, as the L-DOPA dosage increases the therapeutic window (green region) increased (Figure 11).

## Discussion

### MCBG Model

The proposed model tries to present a biologically realistic model of the effect of L-DOPA on PD symptoms, specifically in terms of movement parameters. In our modelling approach, a large-scale cortico-basal ganglia model forms the backbone of our network. The two-link arm model that is interfaced to the MNs simulates the movement of the hand and the feedback related to the hand position and distance from the target is processed by the PC and passed on to MC. MC uses the corrective signals from the BG to initiate the next action. The BG dynamics are highly influenced by the dopaminergic input from the SNc and by incorporating a detailed biophysical model of the SNc into the network model, we were able to show the effect of loss of dopaminergic cells on the movement parameters. Going forward we aim to relate the pathological behaviour with respect to the dynamics at the molecular level happening inside the SNc.

#### Differential Projections and PD Symptoms

The proposed model was able to explain a wide range of pathological behaviours associated with PD by controlling the release of dopamine into the extracellular space and reducing the complexity of the STN-GPe network. We modelled differential projections from the SNc to the Striatum as well as from SNc to STN. By reducing the supply of dopamine through the SNc to Striatum projections, the slowness of movement or bradykinesia could be simulated, and in combination with modulating the complexity of the STN-GPe network through the SNc to STN projections, symptoms like tremor and rigidity were simulated. The complexity of the STN-GPe network was varied by controlling the dopaminergic projections of the SNc neurons toward the STN, thereby affecting the lateral connections within the STN subsystem. By progressively reducing the number of dopaminergic cells in SNc, we could replicate some of the cardinal symptoms of PD–bradykinesia, tremor, and rigidity.

#### L-DOPA Medication Effect

Once the PD condition and the associated symptoms were simulated, we integrated a pharmacokinetic-pharmacodynamic (PK-PD) model of L-DOPA medication (Baston et al., 2016; Véronneau-Veilleux et al., 2020), which showed improved results in reaching performance. L-DOPA medication is one of the first-line treatment methodologies for Parkinson's disease (Suzuki et al., 2020). Our model incorporates the medication effect by interfacing the SNc with the PK-PD model of L-DOPA drug administration. Depending on the dosage of the drug administered, L-DOPA is absorbed into the blood. After interacting with other bodily fluids, a portion of the L-DOPA crosses the BBB and gets absorbed by the dopaminergic terminals. Our results show that consumption of L-DOPA improves the PD symptoms to a great extent. Using our model, we could also see that the extent of improvement on the PD condition depend on the dosage (Figure 12).

A higher level of serum L-DOPA results in dyskinesias and a low-level result in wearing off. Hence, an optimum dosage of medication has to be selected. In order to optimise the drug dosage, we performed our tests with various dosages of L-DOPA medication. We could see that as the percentage of SNc cell loss increases, a higher dosage of L-DOPA was required to sustain the medication effect. With the increase in the percentage of SNc cell loss, the therapeutic effect keeps decreasing. Hence our study focused on the variation of therapeutic effect with respect to the varying percentage SNc cell loss and L-DOPA dosage. The results observed are promising enough to suggest optimal tuning strategies of drug dosage for PD patients (Figure 12). The performance characteristics with respect to the variation in cell loss and the dosage help us to tune the optimum dosage in terms of the quantity and the frequency of dosage.

#### Side-Effects of L-DOPA Medication

From the simulation results, we can explain the L-DOPA wearing-off mechanism to a great extent. Our hypothesis is that the natural progression of the disease characterised by the increase in loss of SNc cells is one of the mechanisms that contribute to L-DOPA wearing off. There could be other factors as well that can accelerate this wearing-off phenomenon. Another hypothesis is that the loss of dopaminergic terminals will lead to synchronised activity in STN which in turn causes overexcitation of SNc neurons resulting in a phenomenon called excitotoxicity in SNc (Muddapu et al., 2019; Muddapu and Chakravarthy, 2020). Thus, fewer dopaminergic terminals and higher L-DOPA dosage result in an accelerated loss of the dopaminergic terminals leading to a faster wearing-off. There might be other contributing factors as well that may advance the shortening of the therapeutic window. There is the potential scope of carrying out a detailed study on the various causes of the L-DOPA wearing off and we believe our model serves as a good platform to conduct such comprehensive research. As shown in our results indicated in Figure 12, we also observe a decrease in performance and reduction in the size of the therapeutic window with an increase in LDOPA dosage beyond a certain value. For example, we can observe the plots of Figure 12A, for 37% cell loss, and Figure 12B, for both 37 and 50% cell loss that if LDOPA dosage is increased beyond 250 mg, the therapeutic window reduces. This reduction in performance can be attributed to Dyskinesias. Hence this model simulation also helps us to optimise the drug dosage with respect to the severity of the disease and the dosage of medication.

The comparison of previous computational models of BG were shown in the Table 2. We can see that the proposed model covers almost all aspects including simulating the PD condition and Behavioural outcomes. It is able to simulate all the cardinal symptoms of TD except the postural imbalance. It is more biologically plausible as a detailed model of SNc for DA release is used and it can simulate the medication effect. Compared to the other models mentioned the current model proposed is having better coverage.

**Table 2:** Comparison of previous computational models for simulating PD behaviour.

**Model**	**PD condition**	**Behavioural output**	**Cardinal symptoms simulation**	**Detailed behavioural model**	**Biological plausibility of DA release**	**Medication effect**
Magdoom et al. (2011)	Yes	Arm reaching	Bradykinesia, tremor, rigidity	No	No	No
Baston et al. (2016)	Yes	Finger tap	Bradykinesia	No	Yes	Yes
Muralidharan et al. (2018)	Yes	Arm reaching	Bradykinesia, tremor, rigidity	Yes	No	No
MCBG	Yes	Arm reaching	Bradykinesia, tremor, rigidity	Yes	Yes	Yes

### Future Scope

We could reliably replicate some of the cardinal symptoms of PD using our MCBG model. Along with simulating the PD ON/OFF mechanisms, our model could also successfully demonstrate the medication effect of L-DOPA. With the L-DOPA PK-PD model integration with the MCBG model, we could also explain the side effects of L-DOPA medication such as dyskinesias and wearing off. Hence this model has the scope to be developed into a test bench for PD. The current diagnostics and treatment methodologies for PD are based on direct observation and therefore suffer from subjectivity (Nair et al., 2022) and we believe that our model can be developed further to provide a more quantitative approach to diagnose PD symptoms and optimise therapeutic interventions.

#### Understanding Causes of Wearing off Mechanism and Dyskinesias

We hypothesise that the natural progression of the disease and the excitotoxicity could be potential factors that result in L-DOPA wearing off. An increase in cytosolic DA will lead to excitotoxicity as unregulated cytosolic DA leads to neurodegeneration (Chen et al., 2008). In this line, the pharmacological model can be extended by incorporating the administration of other drugs that block the vesicular transporter (Pregeljc et al., 2020). In addition to dopamine-induced excitotoxicity, L-DOPA-induced toxicity can also cause neurodegeneration (Fahn, 2005; Lipski et al., 2011; Witt and Fahn, 2016; Muddapu et al., 2020b). However, there could be other contributing factors too and this model can serve as a starting step to explore research in a similar direction. As highlighted in the discussion section, a more detailed study of the L-DOPA wearing-off mechanism can be carried out to understand the mechanism and devise alternate or improved medication regimes. Another line of extension is to explore the phenomenon of different types of dyskinesias such as peak dosage and diphasic dyskinesias (Kim Y. E. et al., 2019).

#### Incorporating DBS to Address Dyskinesias

We also want to extend the model to show the effect of deep brain stimulation (DBS) on motor deficiencies in PD condition and explore the comorbidity effects of both L-DOPA and DBS on PD motor symptoms (Muthuraman et al., 2018; Muddapu et al., 2019; Muddapu and Chakravarthy, 2020; Mueller et al., 2020). One of the limitations of our model is that our model does not consider the influence of the hyperdirect pathway, which involves direct cortical connections to the STN (Nambu et al., 2002; Cai et al., 2019). Also, the model does not take into consideration the influence of cholinergic interneurons in the striatum (Crossley et al., 2016; Kim T. et al., 2019). These can be considered as further enhancements to the current model. Currently, our model is focusing on the motor deficiencies in the PD pathology. It would be interesting to model PD non-motor symptoms (Goldman and Postuma, 2014; Goldman and Guerra, 2020).

## Conclusion

A comprehensive test bench for demonstrating the effect of drug action on symptoms can be a powerful tool in the therapeutic toolkit of neurodegenerative diseases such as Parkinson's disease. Our model is the first step toward this bigger goal. In the current study, we were able to successfully simulate the relationship between drug dosage, cell loss, and PD ON and OFF conditions. We could also demonstrate some of the cardinal symptoms of PD. We also integrated a PK-PD model of L-DOPA medication, which enabled us to simulate the medication effects of the L-DOPA. We also simulated various combinations of L-DOPA medication and percentage of SNc cell loss which enabled us to understand the general trends in drug effects. These modelling results have the potential to optimise the medication in terms of the amount of dosage and the frequency of dosage.

## Data Availability Statement

The raw data supporting the conclusions of this article will be made available by the authors, without undue reservation. Further, inquires can be directed to the corresponding author. The MATLAB code of the proposed MCBG model (http://modeldb.yale.edu/266907) is available on the ModelDB server (McDougal et al., 2017) and an access code will be provided on request.

## Author Contributions

SN, VM, and VC: conceptualization, model development, data curation, formal analysis, investigation, methodology, and validation. SN and VM: visualization and writing—original draft. VC: writing—review, editing, and supervision. All authors contributed to the article and approved the submitted version.

## Funding

The authors would like to thank MHRD, Govt. of India for the HTRA scholarship for Ph.D. students and Center for Complex Systems and Dynamics at IIT Madras for their funding contribution towards the publication fees.

## Conflict of Interest

The authors declare that the research was conducted in the absence of any commercial or financial relationships that could be construed as a potential conflict of interest.

## Publisher's Note

All claims expressed in this article are solely those of the authors and do not necessarily represent those of their affiliated organizations, or those of the publisher, the editors and the reviewers. Any product that may be evaluated in this article, or claim that may be made by its manufacturer, is not guaranteed or endorsed by the publisher.

## References

[B1] Anilkumar U. Khacho M. Cuillerier A. Harris R. Patten D. A. Bilen M.. (2020). MCL-1Matrix maintains neuronal survival by enhancing mitochondrial integrity and bioenergetic capacity under stress conditions. Cell Death Dis. 11, 1–12. 10.1038/s41419-020-2498-9 32371858 PMC7200794

[B2] Armstrong M. J. Okun M. S. (2020). Diagnosis and treatment of Parkinson disease: a review. JAMA 323, 548–560. 10.1001/jama.2019.22360 32044947

[B3] Bakshi S. Chelliah V. Chen C. van der Graaf P. H. (2019). Mathematical biology models of Parkinson's disease. CPT Pharmacomet. Syst. Pharmacol. 8, 77–86. 10.1002/psp4.12362 30358157 PMC6389348

[B4] Balestrino R. Schapira A. H. V. (2020). Parkinson disease. Eur. J. Neurol. 27, 27–42. 10.1111/ene.14108 31631455

[B5] Baston C. Contin M. Buonaura G. C. Cortelli P. Ursino M. (2016). A mathematical model of levodopa medication effect on basal ganglia in parkinson's Disease: an application to the alternate finger tapping task. Front. Hum. Neurosci. 10:280. 10.3389/fnhum.2016.00280 27378881 PMC4911387

[B6] Bereczki D. (2010). The description of all four cardinal signs of Parkinson's disease in a Hungarian medical text published in 1690. Park. Relat. Disord. 16, 290–293. 10.1016/j.parkreldis.2009.11.006 19948422

[B7] Best J. A. Nijhout H. F. Reed M. C. (2009). Homeostatic mechanisms in dopamine synthesis and release: a mathematical model. Theor. Biol. Med. Model. 6, 1–20. 10.1186/1742-4682-6-21 19740446 PMC2755466

[B8] Bogacz R. Gurney K. (2007). The basal ganglia and cortex implement optimal decision making between alternative actions. Neural Comput. 19, 442–447. 10.1162/neco.2007.19.2.442 17206871

[B9] Cai W. Duberg K. Padmanabhan A. Rehert R. Bradley T. Carrion V.. (2019). Hyperdirect insula-basal-ganglia pathway and adult-like maturity of global brain responses predict inhibitory control in children. Nat. Commun. 10:4798. 10.1038/s41467-019-12756-8 31641118 PMC6805945

[B10] Chakravarthy V. S. Balasubramani P. P. (2014). “Basal ganglia system as an engine for exploration,” in Encyclopedia of Computational Neuroscience, eds D. Jaeger and R. Jung (New York, NY: Springer), 1–15. 10.1007/978-1-4614-7320-6_81-1

[B11] Chakravarthy V. S. Moustafa A. A. (2018). Computational Neuroscience Models of the Basal Ganglia. Singapore: Springer Singapore. 10.1007/978-981-10-8494-2

[B12] Chen L. Ding Y. Cagniard B. Van Laar A. D. Mortimer A. Chi W.. (2008). Unregulated cytosolic dopamine causes neurodegeneration associated with oxidative stress in mice. J. Neurosci. 28, 425–433. 10.1523/JNEUROSCI.3602-07.2008 18184785 PMC6670521

[B13] Crossley M. J. Horvitz J. C. Balsam P. D. Ashby F. G. (2016). Expanding the role of striatal cholinergic interneurons and the midbrain dopamine system in appetitive instrumental conditioning. J. Neurophysiol. 115, 240–254. 10.1152/jn.00473.2015 26467514 PMC4760501

[B14] Dorsey E. R. Sherer T. Okun M. S. Bloemd B. R. (2018). The emerging evidence of the Parkinson pandemic. J. Parkinsons. Dis. 8, S3–S8. 10.3233/JPD-181474 30584159 PMC6311367

[B15] Doya K. (1999). What are the computations of the cerebellum, the basal ganglia and the cerebral cortex? Neural Netw. 12, 961–974. 10.1016/S0893-6080(99)00046-5 12662639

[B16] Fahn S. (2005). Does levodopa slow or hasten the rate of progression of Parkinson's disease? J. Neurol. 252, iv37–iv42. 10.1007/s00415-005-4008-5 16222436

[B17] Fitts P. M. (1954). The information capacity of the human motor system in controlling the amplitude of movement. J. Exp. Psychol. 47, 381–391. 10.1037/h0055392 13174710

[B18] Fu H. Hardy J. Duff K. E. (2018). Selective vulnerability in neurodegenerative diseases. Nat. Neurosci. 21, 1350–1358. 10.1038/s41593-018-0221-2 30250262 PMC6360529

[B19] Fullard M. E. Morley J. F. Duda J. E. (2017). Olfactory dysfunction as an early biomarker in Parkinson's disease. Neurosci. Bull. 33, 515–525. 10.1007/s12264-017-0170-x 28831680 PMC5636737

[B20] Giguère N. Delignat-Lavaud B. Herborg F. Voisin A. Li Y. Jacquemet V.. (2019). Increased vulnerability of nigral dopamine neurons after expansion of their axonal arborization size through D2 dopamine receptor conditional knockout. PLOS Genet. 15:, e1008352. 10.1371/journal.pgen.1008352 31449520 PMC6730950

[B21] Gillies A. Willshaw D. Atherton J. Arbuthnott G. (2002). “Functional interactions within the subthalamic nucleus,” in Brain (Oxford University Press), 359–368. 10.1007/978-1-4615-0715-4_36

[B22] Goldman J. G. Guerra C. M. (2020). Treatment of nonmotor symptoms associated with Parkinson disease. Neurol. Clin. 38, 269–292. 10.1016/j.ncl.2019.12.003 32279710

[B23] Goldman J. G. Postuma R. (2014). Premotor and nonmotor features of Parkinson's disease. Curr. Opin. Neurol. 27, 434–441. 10.1097/WCO.0000000000000112 24978368 PMC4181670

[B24] Gonzalez-Rodriguez P. Zampese E. Surmeier D. J. (2020). Selective neuronal vulnerability in Parkinson's disease. Prog. Brain Res. 252, 61–89. 10.1016/bs.pbr.2020.02.005 32247375

[B25] Gurney K. Prescott T. J. Redgrave P. (2001). A computational model of action selection in the basal ganglia. I. A new functional anatomy. Biol. Cybern. 84, 401–410. 10.1007/PL00007984 11417052

[B26] Hikosaka O. Nakamura K. Sakai K. Nakahara H. (2002). Central mechanisms of motor skill learning. Curr. Opin. Neurobiol. 12, 217–222. 10.1016/S0959-4388(02)00307-0 12015240

[B27] Humphries M. D. Stewart R. D. Gurney K. N. (2006). A physiologically plausible model of action selection and oscillatory activity in the basal ganglia. J. Neurosci. 26, 12921–12942. 10.1523/JNEUROSCI.3486-06.2006 17167083 PMC6674973

[B28] Izawa J. Kondo T. Ito K. (2004). Biological arm motion through reinforcement learning. Biol. Cybern. 91, 10–22. 10.1007/s00422-004-0485-3 15309543

[B29] Jagodnik K. M. van den Bogert A. J. (2010). Optimization and evaluation of a proportional derivative controller for planar arm movement. J. Biomech. 43, 1086–1091. 10.1016/j.jbiomech.2009.12.017 20097345 PMC3853125

[B30] Kawahara T. (1980). Coupled Van der Pol oscillators? A model of excitatory and inhibitory neural interactions. Biol. Cybern. 39, 37–43. 10.1007/BF00336943 7459399

[B31] Kim T. Capps R. A. Hamade K. C. Barnett W. H. Todorov D. I. Latash E. M.. (2019). The functional role of striatal cholinergic interneurons in reinforcement learning from computational perspective. Front. Neural Circuits 13:10. 10.3389/fncir.2019.00010 30846930 PMC6393383

[B32] Kim Y. E. Jeon B. Yun J. Y. Yang H. J. Kim H. J. (2019). Chronological view of peak and diphasic dyskinesia, wearing off and freezing of gait in Parkinson's disease. J. Parkinsons. Dis. 9, 741–747. 10.3233/JPD-191624 31498128

[B33] Knill D. C. Pouget A. (2004). The Bayesian brain: the role of uncertainty in neural coding and computation. Trends Neurosci. 27, 712–719. 10.1016/j.tins.2004.10.007 15541511

[B34] Kohonen T. (2001). Self-Organizing Maps, 3rd edn. Berlin: Springer-Verlag Berlin Heidelberg.

[B35] Körding K. P. Wolpert D. M. (2004). Bayesian integration in sensorimotor learning. Nature 427, 244–247. 10.1038/nature02169 14724638

[B36] Lipski J. Nistico R. Berretta N. Guatteo E. Bernardi G. Mercuri N. B. (2011). L-DOPA: a scapegoat for accelerated neurodegeneration in Parkinson's disease? Prog. Neurobiol. 94, 389–407. 10.1016/j.pneurobio.2011.06.005 21723913

[B37] Magdoom K. N. Subramanian D. Chakravarthy V. S. Ravindran B. Amari S. Meenakshisundaram N. (2011). Modeling basal ganglia for understanding parkinsonian reaching movements. Neural Comput. 23, 477–516. 10.1162/NECO_a_00073 21105828

[B38] Majsak M. J. Kaminski T. Gentile A. M. Flanagan J. R. (1998). The reaching movements of patients with Parkinson's disease under self-determined maximal speed and visually cued conditions. Brain 121, 755–766. 10.1093/brain/121.4.755 9577399

[B39] Marino B. L. B. de Souza L. R. Sousa K. P. A. Ferreira J. V. Padilha E. C. da Silva C. H. T. P.. (2020). Parkinson's disease: a review from pathophysiology to treatment. Mini-Reviews Med. Chem. 20, 754–767. 10.2174/1389557519666191104110908 31686637

[B40] Marras C. Beck J. C. Bower J. H. Roberts E. Ritz B. Ross G. W.. (2018). Prevalence of Parkinson's disease across North America. NPJ Park. Dis. 4, 1–7. 10.1038/s41531-018-0058-0 30003140 PMC6039505

[B41] McDougal R. A. Morse T. M. Carnevale T. Marenco L. Wang R. Migliore M.. (2017). Twenty years of ModelDB and beyond: building essential modeling tools for the future of neuroscience. J. Comput. Neurosci. 42, 1–10. 10.1007/s10827-016-0623-7 27629590 PMC5279891

[B42] Michel P. P. Hirsch E. C. Hunot S. (2016). Understanding dopaminergic cell death pathways in Parkinson disease. Neuron 90, 675–691. 10.1016/j.neuron.2016.03.038 27196972

[B43] Morasso P. (1981). Spatial control of arm movements. Exp. Brain Res. 42, 223–227. 10.1007/BF00236911 7262217

[B44] Morley J. F. Duda J. E. (2010). Olfaction as a biomarker in Parkinsons disease. Biomark. Med. 4, 661–670. 10.2217/bmm.10.95 20945979

[B45] Moustafa A. A. Chakravarthy V. S. Phillips J. R. Crouse J. J. Gupta A. Frank M. J.. (2016). Interrelations between cognitive dysfunction and motor symptoms of Parkinson's disease: behavioral and neural studies. Rev. Neurosci. 27, 535–548. 10.1515/revneuro-2015-0070 26982614

[B46] Muddapu V. R. Chakravarthy V. S. (2020). A multi-scale computational model of excitotoxic loss of dopaminergic cells in Parkinson's disease. Front. Neuroinform. 14:34. 10.3389/fninf.2020.00034 33101001 PMC7555610

[B47] Muddapu V. R. Chakravarthy V. S. (2021). Influence of energy deficiency on the subcellular processes of substantia nigra pars compacta cell for understanding parkinsonian neurodegeneration. Sci. Rep. 11:1754. 10.1038/s41598-021-81185-9 33462293 PMC7814067

[B48] Muddapu V. R. Dharshini S. A. P. Chakravarthy V. S. Gromiha M. M. (2020a). Neurodegenerative diseases – is metabolic deficiency the root cause? Front. Neurosci. 14:213. 10.3389/fnins.2020.00213 32296300 PMC7137637

[B49] Muddapu V. R. Mandali A. Chakravarthy V. S. Ramaswamy S. (2019). A computational model of loss of dopaminergic cells in Parkinson's disease due to glutamate-induced excitotoxicity. Front. Neural Circuits 13:11. 10.3389/fncir.2019.00011 30858799 PMC6397878

[B50] Muddapu V. R. Vijaykumar K. Ramakrishnan K. Chakravarthy V. S. (2020b). A computational model of levodopa-induced toxicity in substantia nigra pars compacta in Parkinson's disease. bioRxiv, 1–55. 10.1101/2020.04.05.026807

[B51] Mueller K. Urgošík D. Ballarini T. Holiga Š. Möller H. E. RuŽička F.. (2020). Differential effects of deep brain stimulation and levodopa on brain activity in Parkinson's disease. Brain Commun. 2:fcaa005. 10.1093/braincomms/fcaa005 32954278 PMC7425344

[B52] Muralidharan V. Mandali A. Balasubramani P. P. Mehta H. Srinivasa Chakravarthy V. Jahanshahi M. (2018). “A cortico-basal ganglia model to understand the neural dynamics of targeted reaching in normal and parkinson's conditions,” in Computational Neuroscience Models of the Basal Ganglia, eds V. S. Chakravarthy and A. A. Moustafa (Singapore: Springer Singapore), 167–195. 10.1007/978-981-10-8494-2_10

[B53] Muthuraman M. Koirala N. Ciolac D. Pintea B. Glaser M. Groppa S.. (2018). Deep brain stimulation and L-DOPA therapy: concepts of action and clinical applications in parkinson's disease. Front. Neurol. 9:711. 10.3389/fneur.2018.00711 30210436 PMC6119713

[B54] Nair S. S. Muddapu V. R. J. Sriram M. Aditya R. Gupta R. Chakravarthy S. (2022). “Is There a Better Way to Assess Parkinsonian Motor Symptoms?–Experimental and Modelling Approach,” in Techniques for Assessment of Parkinsonism for Diagnosis and Rehabilitation: Series in BioEngineering, eds S. P. Arjunan and D. K. Kumar (Singapore: Springer). 10.1007/978-981-16-3056-9_10

[B55] Nakahara H. Doya K. Hikosaka O. (2001). Parallel cortico-basal ganglia mechanisms for acquisition and execution of visuomotor sequences - a computational approach. J. Cogn. Neurosci. 13, 626–647. 10.1162/089892901750363208 11506661

[B56] Nambu A. Tokuno H. Takada M. (2002). Functional significance of the cortico-subthalamo-pallidal “hyperdirect” pathway. Neurosci. Res. 43, 111–117. 10.1016/S0168-0102(02)00027-5 12067746

[B57] Narayanamurthy R. Jayakumar S. Elango S. Muralidharan V. Chakravarthy V. S. (2019). A cortico-basal ganglia model for choosing an optimal rehabilitation strategy in hemiparetic stroke. Sci. Rep. 9:13472. 10.1038/s41598-019-49670-4 31530821 PMC6748960

[B58] Nomoto M. Nagai M. Nishikawa N. Ando R. Kagamiishi Y. Yano K.. (2018). Pharmacokinetics and safety/efficacy of levodopa pro-drug ONO-2160/carbidopa for Parkinson's disease. eNeurologicalSci 13, 8–13. 10.1016/j.ensci.2018.09.003 30294682 PMC6171046

[B59] Pacelli C. Giguère N. Bourque M.-J. Lévesque M. Slack R. S. Trudeau L.-É. (2015). Elevated mitochondrial bioenergetics and axonal arborization size are key contributors to the vulnerability of dopamine neurons. Curr. Biol. 25, 2349–2360. 10.1016/j.cub.2015.07.050 26320949

[B60] Pissadaki E. K. Bolam J. P. (2013). The energy cost of action potential propagation in dopamine neurons: clues to susceptibility in Parkinson's disease. Front. Comput. Neurosci. 7:13. 10.3389/fncom.2013.00013 23515615 PMC3600574

[B61] Poewe W. Seppi K. Tanner C. M. Halliday G. M. Brundin P. Volkmann J.. (2017). Parkinson disease. Nat. Rev. Dis. Prim. 3, 1–21. 10.1038/nrdp.2017.13 28332488

[B62] Pregeljc D. Teodorescu-Perijoc D. Vianello R. Umek N. Mavri J. (2020). How important is the use of cocaine and amphetamines in the development of Parkinson disease? A computational st*ud*y. Neurotox. Res. 37, 724–731. 10.1007/s12640-019-00149-0 31828739

[B63] Reed M. C. Nijhout H. F. Best J. A. (2012). Mathematical insights into the effects of levodopa. Front. Integr. Neurosci. 6:21. 10.3389/fnint.2012.00021 22783173 PMC3389445

[B64] Schultz W. (1998). Predictive reward signal of dopamine neurons. J. Neurophysiol. 80, 1–27. 10.1152/jn.1998.80.1.1 9658025

[B65] Shadmehr R. Krakauer J. W. (2008). A computational neuroanatomy for motor control. Exp. Brain Res. 185, 359–381. 10.1007/s00221-008-1280-5 18251019 PMC2553854

[B66] Shanbhag T. Shenoy S. (2020). Pharmacology for Medical Graduates, 4th edn. New Delhi: Elsevier India.

[B67] Sridharan D. Prashanth P. S. Chakravarthy V. S. (2006). The role of the basal ganglia in exploration in A neural model based on reinforcement learning. Int. J. Neural Syst. 16, 111–124. 10.1142/S0129065706000548 16688851

[B68] Surmeier D. J. (2018). Determinants of dopaminergic neuron loss in Parkinson's disease. FEBS J. 285, 3657–3668. 10.1111/febs.14607 30028088 PMC6546423

[B69] Suzuki M. Arai M. Hayashi A. Ogino M. (2020). Adherence to treatment guideline recommendations for Parkinson's disease in Japan: a longitudinal analysis of a nationwide medical claims database between 2008 and 2016. PLoS ONE 15:e0230213. 10.1371/journal.pone.0230213 32330133 PMC7182259

[B70] Todorov E. (2004). Optimality principles in sensorimotor control. Nat. Neurosci. 7, 907–915. 10.1038/nn1309 15332089 PMC1488877

[B71] Trappenberg T. P. (2005). “Continuous Attractor Neural Networks,” in Recent Developments in Biologically Inspired Computing, eds L. N. de Castro and F. J. Vonzubin (New York, NY: IGI Global), 398–425. 10.4018/978-1-59140-312-8.ch016

[B72] Véronneau-Veilleux F. Ursino M. Robaey P. Lévesque D. Nekka F. (2020). Nonlinear pharmacodynamics of levodopa through Parkinson's disease progression. Chaos 30:093146. 10.1063/5.0014800 33003902

[B73] Witt P. A. L. Fahn S. (2016). Levodopa therapy for Parkinson disease: a look backward and forward. Neurology 86, S3–S12. 10.1212/WNL.0000000000002509 27044648

[B74] Zadravec M. Matjačić Z. (2013). Planar arm movement trajectory formation: an optimization based simulation study. Biocybern. Biomed. Eng. 33, 106–117. 10.1016/j.bbe.2013.03.006

